# Single cell analysis of developing Merkel cells reveals the emergence of non-coding RNA biotypes as a hallmark of terminal differentiation

**DOI:** 10.1038/s41418-026-01663-3

**Published:** 2026-02-03

**Authors:** Lingling Miao, Loren Collado, Savannah Barkdull, Patrick Hallaert, Mackenzie R. Martin, Berkley E. Gryder, Michael C. Kelly, Stefania Dell’Orso, Matthew W. Kelley, Isaac Brownell

**Affiliations:** 1 Dermatology Branch, National Institute of Arthritis and Musculoskeletal and Skin Diseases, National Institutes of Health, Bethesda, MD USA; 2 Department of Genetics and Genome Sciences, Case Western Reserve University, Cleveland, OH USA; 3 Laboratory of Cochlear Development, National Institute on Deafness and Other Communication Disorders, National Institutes of Health, Bethesda, MD USA; 4 Genomic Technology Section, National Institute of Arthritis and Musculoskeletal and Skin Diseases, National Institutes of Health, Bethesda, MD USA

**Keywords:** Development, Gene expression, Gene regulation

## Abstract

RNA processing generates diverse protein-coding and non-coding transcripts, yet RNA biotype diversity during cellular differentiation is not well characterized. Merkel cells (MCs) are cutaneous mechanosensors. We analyzed full-length transcripts of FACS-sorted single mouse MCs at all stages of development and discovered that their terminal differentiation was accompanied by an emergence of non-coding transcripts associated with genes related to MC function. Non-coding RNAs upregulated during terminal differentiation included retained intron transcripts capable of forming nuclear condensates that contained their cognate mRNAs. We showed that *Aspa* retained intron condensates prevented the nuclear export of *Aspa* mRNA, reducing ASPA expression. Transcripts associated with terminal differentiation in five other mammalian cell types also showed an increased abundance of non-coding biotypes and this was attenuated in differentiation-defective Down syndrome neurons. These findings strongly suggest that the emergence of non-coding transcripts is a general feature of terminal differentiation and retained intron condensates can function to regulate gene expression.

## Introduction

Transcription of eukaryotic genomes and post-transcriptional RNA processing, such as alternative splicing, generates diverse RNA species, including protein-coding and non-coding transcript biotypes [1–3]. In addition to canonical mRNA isoforms, regulated splicing also produces transcript biotypes such as those with retained introns or those channeled to nonsense-mediated decay (NMD) [4–6]. Non-coding RNA biotypes increase the diversity and complexity of eukaryotic transcriptomes. In many cases, these non-coding biotypes play important roles in regulating gene expression [7–11]. Although individual splicing factors and non-coding transcripts have been reported to regulate aspects of development, global changes in RNA biotype diversity during cellular differentiation are not yet fully characterized.

Single-cell RNA sequencing (scRNA-seq) using SMART (Switching Mechanism at the 5′ end of RNA Template) technology library preparation has the potential to capture full-length transcripts and generate deep sequencing data appropriate for transcript-level analysis and the temporal modelling of cellular differentiation [12, 13]. Recent studies have demonstrated heterogeneity in transcript isoform expression among single cells, developmentally regulated alternative splicing events, and dynamic expression of splicing factors and RNA-binding proteins during cell development [14–17]. Unique non-coding RNA expression patterns have also been associated with cell cycle and developmental states [18]. Studying single-cell transcriptomes to understand the protein-coding and non-coding transcript landscape is needed to fully explore cell state changes during differentiation.

Merkel cells (MCs) are neuroendocrine cells found in the basal layer of skin that are innervated by sensory nerve endings and function in light touch sensation [19]. We analyzed the transcriptomes of differentiating MCs using Smart-seq2 scRNA-seq to map their developmental trajectory. Our findings revealed an emergence of non-coding transcripts, including retained intron (RI) transcripts, during terminal differentiation. Some of these RI transcripts acted as post-transcriptional regulators by forming nuclear condensates that sequestered their cognate mRNAs and downregulated protein expression. Analysis of other differentiating cell types confirmed that the emergence of non-coding transcript biotypes is a hallmark of terminal differentiation. Additionally, neurons from patients with Down syndrome (DS) showed reduced non-coding transcript emergence, consistent with impaired neuronal maturation.

## Results

### FACS-based scRNA-seq identifies trajectory for mouse MC differentiation

**Fig. 1:**
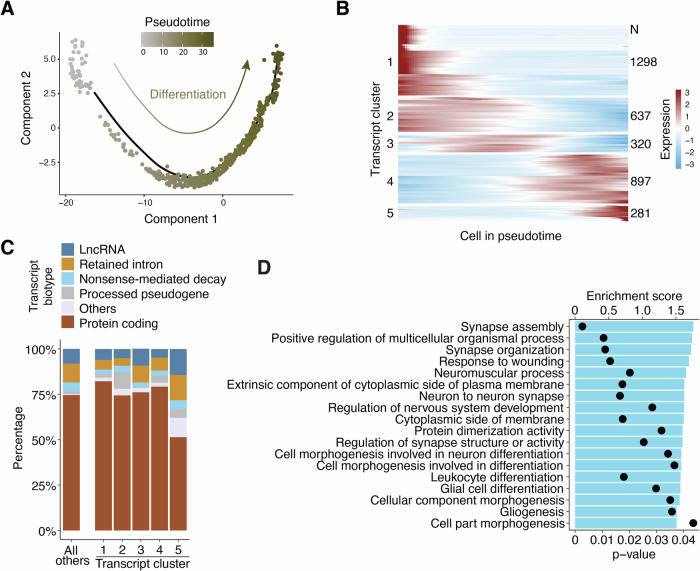
MC terminal differentiation is accompanied by emergence of non-protein coding transcript biotypes associated with genes related to cell specific function. **A** Monocle-generated trajectory plot representing differentiation pseudotime ordering of single MCs. **B** Scaled and centered expression heatmap of transcripts with dynamic expression over the MC differentiation pseudotime. Transcripts are ordered by row and cells by column. Transcripts are clustered by their expression patterns over pseudotime. The number of transcripts per cluster is shown. **C** Bar chart showing the relative proportion of transcript biotypes in each pseudotime-dependent transcript clusters from (**B**) and all other transcripts. All others: 13,600 detected transcripts with TPM > 10 in more than 58 MCs and not included among the dynamically expressed transcripts. Chi-squared test: *p* < 2.2 × 10^−16^ for protein-coding vs. non-coding transcript numbers across all clusters, including “All others”. **D** Top gene ontology terms associated with genes encoding non-coding transcript biotypes in transcript cluster 4 and 5 from (**B**) (highest expression in late differentiation/terminally differentiated MCs). Bar magnitude, enrichment score; dot, *p*-value. See also Supplementary Figs. 1 and 2.

Sensory MCs in mouse skin express SOX2 and GFI1 [20–22]. To define the full trajectory of MC differentiation, we isolated MCs from both *Sox2*^*GFP/+*^ and *Gfi1*^*GFP/+*^ reporter mice at embryonic (E16.5, E17.5, E18.5), neonatal (P0), and postnatal timepoints (P6) using fluorescence-activated cell sorting (FACS). We prepared single-cell poly-A selected cDNA libraries using the Smart-seq2 method [12], which were then deeply sequenced to yield ~3 million uniquely mapped reads per cell. A total of 698 single MC transcriptomes passed quality control. A trajectory analysis using Monocle [23] ordered single cells along an unbranched pseudotime-line (Fig. 1A). As expected, the cell trajectory was consistent with sampling timepoints (Supplementary Fig. 1A) and MCs from both *Sox2*^*GFP/+*^ and *Gfi1*^*GFP/+*^ reporters were similarly distributed along the pseudotime-line (Supplementary Fig. 1B). To validate the pseudotime axis relative to MC differentiation, we examined the dynamic expression of genes associated with MC progenitors (Supplementary Fig. 1C) and differentiated MCs (Supplementary Fig. 1D). Expression of progenitor markers such as *Krt5*, *Krt14*, and *Krt17* decreased rapidly early on in differentiation (Supplementary Fig. 1C). Conversely, markers of differentiating MCs such as *Atoh1, Krt8*, and *Krt18* increased expression over the trajectory. Finally, mature MC genes such as *Piezo2*, *Krt20*, and *Rab3b* were all highly expressed later in the trajectory (Supplementary Fig. 1D).

### MC terminal differentiation is accompanied by the emergence of non-protein coding transcript biotypes associated with functionally relevant genes

The Smart-seq2 library preparation method enables the capture of full-length cDNA transcriptomes. We used RSeQC to determine the gene body overage (distribution of reads along transcripts) in single MCs based on housekeeping genes and found that our scRNA-seq reads covered transcripts uniformly (Supplementary Fig. 2A). Using the intron-aware transcript aligner STAR for mapping and RSEM for quantification, we generated a transcript expression matrix containing the abundance of individual RNA transcripts in single MCs. We performed trajectory analysis based on transcript expression, and the resulting pseudotime ordering of single MCs was highly correlated to the pseudotime-line based on gene expression (*r* = 0.98, Supplementary Fig. 2B). Thus, we continued to use our original pseudotime-line for consistency. Monocle analysis identified 3433 transcripts with dynamic expression during MC differentiation, organized into 5 clusters based on expression patterns along the differentiation pseudotime (Fig. 1B). Distinct from the 3433 transcripts, we detected 13,600 additional transcripts expressed with greater than 10 transcripts per million (TPM) in more than 58 single MCs (labeled as “All others”, see Methods for details).

Transcript biotypes were annotated using Ensembl predictions. Approximately 75% of expressed transcripts were annotated as protein-coding. Remaining non-coding transcripts captured in our libraries were predominantly RI, lncRNA, NMD and processed pseudogene biotypes (Fig. 1C). Analyzing the relative composition of annotated biotypes for each pseudotime-dependent transcript cluster, we found that cluster 5 (transcripts upregulated in fully differentiated MCs) contained proportionally fewer protein-coding transcripts (51.4%), compared to other transcript clusters and the global average (Fig. 1C). As non-protein coding transcript biotypes such as RI and NMD can influence mRNA levels of their cognate genes [4, 9], we analyzed genes associated with the non-coding transcripts that increase their expression during MC terminal differentiation (transcript clusters 4 and 5). GO enrichment analysis found that the non-coding transcripts that emerge during MC differentiation were associated with genes highly enriched in neuronal differentiation and function, suggesting their importance in regulating the neurosensory function of MCs (Fig. 1D). In contrast to the dynamically expressed transcripts, the relative expression of protein-coding to non-coding transcripts across all expressed transcripts was consistent throughout MC differentiation (Supplementary Fig. 2C, D), suggesting that the enrichment of non-coding transcripts associated with terminal differentiation is preferentially in genes related to cellular function.

### Retained intron transcripts can form extrachromosomal nuclear condensates

**Fig. 2:**
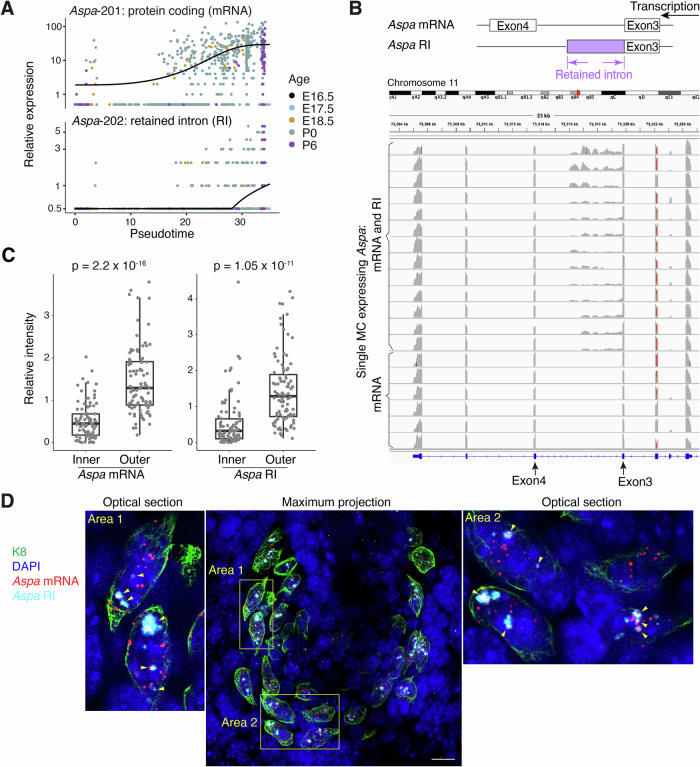
Retained intron transcript *Aspa*-202 is co-expressed with *Aspa* mRNA transcripts in single MCs and forms nuclear condensates. **A** Scatter plot indicating the expression levels of *Aspa*-201: protein-coding (mRNA) and *Aspa*-202: retained intron (RI) transcripts in single MCs along the pseudotime of MC differentiation. **B** Upper: Schematic illustration of the RI sequence between exon 3 and exon 4 in *Aspa*-202 transcript. Lower: IGV-generated plot of sequencing reads aligned to the *Aspa* gene locus indicating RI transcript in select MCs. **C** Boxplot showing the smFISH relative signal intensity of the target transcripts per cell in inner (less differentiated) and outer (more differentiated) neonatal touch dome MCs. Boxplots show median, interquartile range (IQR), and whiskers extending to 1.5 x IQR. Data were collected from 9 touch domes across 3 mice. N (Inner) = 88; N (Outer) = 92. **D** Middle: Confocal z-projection of whole mount smFISH displaying the expression of *Aspa* mRNA (red) and *Aspa* RI (cyan) transcripts in neonatal mouse epidermis. MCs, K8 immunostaining (green). Cell nuclei, DAPI (blue). Left and right: optical sections of representative MCs. Arrowhead indicates colocalization of *Aspa* mRNA and *Aspa* RI. Scale bar, 10μm. See also Supplementary Figs. 3–5.

RI transcripts were highly represented among the non-protein coding biotypes that emerged during MC terminal differentiation. We often detected genes expressing both RI and mRNA transcripts in the same MC. To validate this, we selected genes whose RI and mRNA transcripts had sequence regions that were mutually exclusive and designed probes for single-molecule RNA fluorescence in situ hybridization (smFISH). For example, *Aspa* encodes aspartoacylase, which deacetylates the neuron-specific amino acid derivative N-acetyl-L-aspartic acid to yield aspartate and acetate and has a critical function in the brain [24]. In MCs, *Aspa* expressed mRNA transcripts and *Aspa*-202 RI transcript with partial retention of the intron between exon 3 and exon 4. Both transcripts increased during MC differentiation (Fig. 2A and Supplementary Fig. 3A, B). Within single MC we observe a subset of transcripts with sequence reads aligned to this RI, but not to other intronic regions (Fig. 2B), making it unlikely that we were sequencing unspliced pre-mRNA transcripts. Consistent with increasing transcript expression, read counts specifically mapping to the RI region increased during MC differentiation (Supplementary Fig. 3C). In addition to the *Aspa*-202 RI transcript, our scRNA-seq analyses detected the expression of two mRNA transcripts and one NMD transcript in differentiating MCs (Supplementary Fig. 3B). Using whole mount staining of neonatal mouse epidermis with smFISH probes to detect exons of the *Aspa* mRNAs or the *Apsa*-202 RI (Supplementary Fig. 3A), we detected mRNA and RI transcripts co-expressed in single MCs (Fig. 2D, Supplementary Fig. 4B–D, and Supplementary Video 1). As expected, *Aspa* expression in the skin was restricted to MCs (marked by K8 immunostaining). Interestingly, the mRNA smFISH detected multiple discrete small puncta in the cytoplasm and nuclei of MC with 75% ± 10% (mean ± standard deviation) of the FISH signal localizing within nuclei (Supplementary Fig. 4A). In contrast, RI smFISH signals were fewer, larger, often co-localized around mRNA nuclear puncta, and showed 97% ± 3% nuclear localization (Supplementary Fig. 4A). To better resolve the location of the RI puncta, we stained MCs freshly isolated from P0 mouse skin and found they localized exclusively outside of DAPI-staining regions within nuclei, indicative of them being extrachromosomal nuclear condensates [25] (Supplementary Fig. 3G). In hairy skin, MCs congregate in specialized epidermal structures named touch domes [26]. We previously reported that MCs in the outer P0 touch dome are more mature than those in the inner touch dome [27]. Consistent with their expression in more differentiated MCs (Fig. 2A and Supplementary Fig. 3B), the smFISH intensity and colocalization of the *Aspa* mRNA and RI transcripts were increased in outer touch dome MCs (Fig. 2C and Supplementary Fig. 3D–F). Analysis of *Vwa5b2*, another nerve-expressed gene whose protein-coding and non-coding transcripts increased during MC differentiation, showed analogous read mapping and smFISH results for its mRNA and RI transcripts (Supplementary Fig. 5 and Supplementary Video 2). These results demonstrate that both protein-coding and RI transcripts can be expressed from a gene within a single cell. Moreover, their colocalization patterns suggest RI transcripts may physically surround mRNA transcripts in the nucleus as a possible mechanism for posttranscriptional regulation in differentiating MCs.

### Retained intron transcripts can induce nuclear retention of mRNA and downregulate protein expression

Our smFISH analysis of mouse MCs found that *Aspa* and *Vwa5b2* RI transcripts formed extrachromosomal nuclear condensates that often contained cognate mRNA transcripts. To test our hypothesis that RI condensates sequester mRNA and downregulate gene expression, we employed mouse HT-22 cells. This immortalized neuronal cell line can be terminal differentiated in vitro by changing culture conditions [28].

**Fig. 3:**
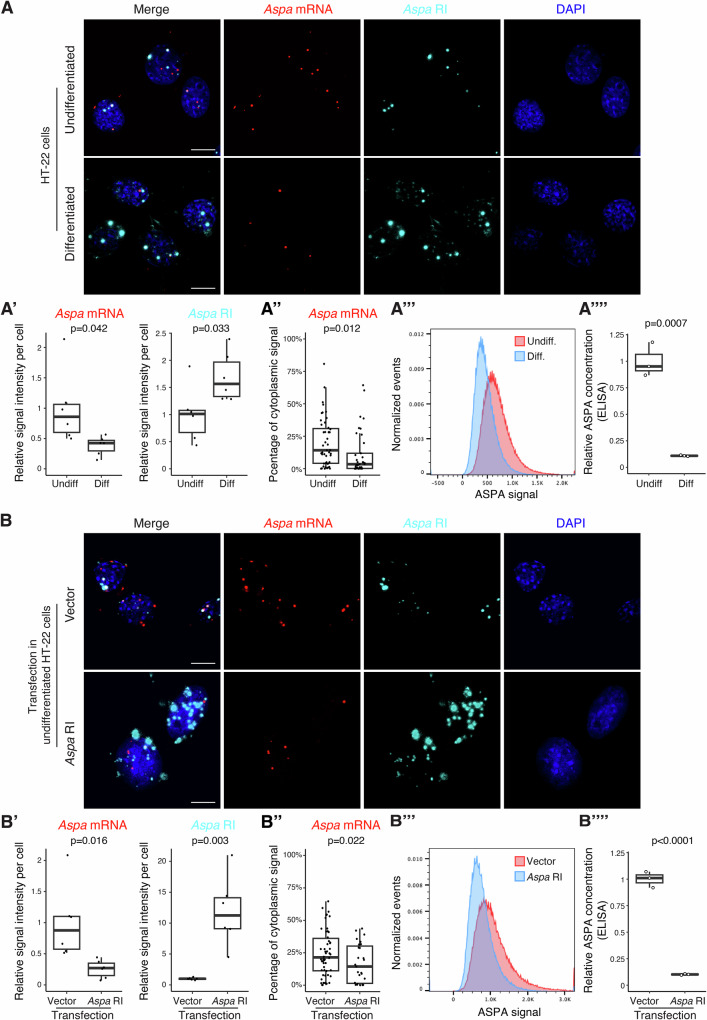
Retained intron transcripts downregulate cognate gene expression. **A** Confocal optical sections of smFISH displaying the expression of *Aspa* mRNA (red) and *Aspa* RI (cyan) transcripts in undifferentiated (upper) and in vitro differentiated (lower) HT-22 cells. Cell nuclei, DAPI (blue). Scale bar, 10 μm. **B** Confocal optical sections of smFISH displaying the expression of *Aspa* mRNA (red) and *Aspa* RI (cyan) transcripts in vector control transfected (upper) and *Aspa* RI transfected (lower) undifferentiated HT-22 cells. Cell nuclei, DAPI (blue). Scale bar, 10 μm. **A’, B’** Boxplot showing the smFISH average relative signal intensity per cell in undifferentiated (undiff) and differentiated (diff) (**A’**), and in vector and *Aspa* RI transfected (**B’**) HT-22 cells. Each dot represents the average signal intensity per cell within a high-power field (range 6-28 cells). Results include cells from 3 independent experiments. *N* = 12 fields. **A”**, **B”** Boxplot showing the cytoplasmic fraction per cell of *Aspa* mRNA smFISH signal intensity in undifferentiated and differentiated (**A”**), and in vector and *Aspa* RI transfected (**B”**) HT-22 cells. Each dot represents an individual cell. Results are combined from 3 independent experiments. N (undifferentiated cells) = 47; N (differentiated cells) = 37; N (vector transfected cells) = 54; N (*Aspa* RI transfected cells) = 27. **A”’**, **B”’**, **A””**, **B””** ASPA protein expression levels in undifferentiated and differentiated (**A”’**, **A””**), and in vector and *Aspa* RI transfected (**B”’**, **B””**) HT-22 cells. (**A”’**, **B”’**), representative flow cytometry histogram showing the normalized ASPA signal events. ASPA mean fluorescence intensity (MFI): differentiated vs. undifferentiated = 0.69 ± 0.05 (*N* = 3, *p* = 0.0006); *Aspa* RI vs. empty vector transfection = 0.73 ± 0.10 (*N* = 5, *p* = 0.0004). (**A””**, **B””**), boxplot showing the median, interquartile range (IQR), and whiskers extending to 1.5 x IQR of ASPA protein concentration relative to control groups, as measured by ELISA (*N* = 3). See also Supplementary Fig. 6-8.

Using smFISH, we confirmed that *Aspa*-201 (mRNA) and *Aspa*-202 (RI) transcripts were co-expressed in HT-22 cells. Consistent with our findings in MCs, *Aspa* RI formed nuclear condensates that colocalized with *Aspa* mRNA (Fig. 3A). Upon in vitro differentiation of HT-22 cells, *Aspa* RI expression increased, whereas *Aspa* mRNA expression was reduced (Fig. 3A, A’). The increase in RI condensates observed during differentiation was accompanied by a reduction in the cytoplasmic fraction of *Aspa* mRNA (Fig. 3A”), indicating mRNA nuclear retention. Flow cytometry after intracellular immunostaining for ASPA demonstrated that protein expression levels were also reduced (Fig. 3A”’). ASPA quantification by ELISA showed an 89.1% reduction in protein levels (Fig. 3A””). These data confirmed, in a second cell type, that terminal differentiation was accompanied by an increase in *Aspa* RI transcripts. Moreover, the data demonstrated that this RI increase is associated with mRNA nuclear retention and reduced protein expression.

To show that increased *Aspa* RI transcripts were responsible for the changes in mRNA localization and protein levels, we overexpressed a full-length *Aspa* RI transcript (*Aspa*-202) in undifferentiated HT-22 cells. We found that similar to terminal differentiation, overexpressing *Aspa* RI transcripts was sufficient to increase nuclear condensate formation (Fig. 3B and Fig. 3B’), decrease the cytoplasmic fraction and expression levels of *Aspa* mRNA (Fig. 3B’ and Fig. 3B”), and resulted in an 89.9% reduction of ASPA protein levels (Fig. 3B”’ and Fig. 3B””). *Aspa* RI overexpression did not lead to global changes in RNA expression (Supplementary Fig. 6), suggesting that the effect of *Aspa* RI is relatively specific to *Aspa* mRNA. Overexpression of *Aspa* RI did not induce phenotypic neuronal differentiation, alter cell apoptosis and cell proliferation, or promote cell cycle exit in HT-22 cells (Supplementary Fig. 7), supporting a direct effect by the RI transcript in reducing ASPA expression as opposed to a secondary consequence of altered cell biology. Consistently, direct *Aspa* knockdown using siRNAs had no detectable effect on HT-22 cell proliferation and differentiation (Supplementary Fig. 8), demonstrating that although *Aspa* expression is modulated during terminal differentiation, it does not appear to be necessary for differentiation. Taken together, these data strongly suggest that RI transcripts can form nuclear condensates capable of regulating the subcellular localization and expression of their cognate mRNAs. This novel regulatory mechanism expands the functional roles of non-coding RNA transcripts in modulating gene expression.

### Emergence of non-protein coding RNA biotypes is a general feature of terminal differentiation

Having found that functionally relevant non-coding transcripts emerge during MC differentiation, we sought to test if this was a property of terminal differentiation in other cell types. We analyzed public full-length scRNA-seq datasets of differentiating tissues/cells obtained using Smart-seq methods: mouse lung alveolar type II cell development [29] (*Sftpc* + AT2 cell linage, “mouse AT2” hereafter), human neurons in vitro differentiated from neural precursor cells (NPCs) [30] (“human neuron” hereafter), mouse neurons and myocytes in vitro differentiated from mouse embryonic fibroblast (MEF) [31] (“mouse neuron” and “mouse myocyte” hereafter) and mouse growth plate differentiation [32] (“mouse GP” hereafter). All these studies used the Smart-seq method for the library preparation and performed sequencing with paired-end runs no shorter than 100 bp. We downloaded raw sequencing data (FASTQ) and analyzed with our pipeline.

**Fig. 4:**
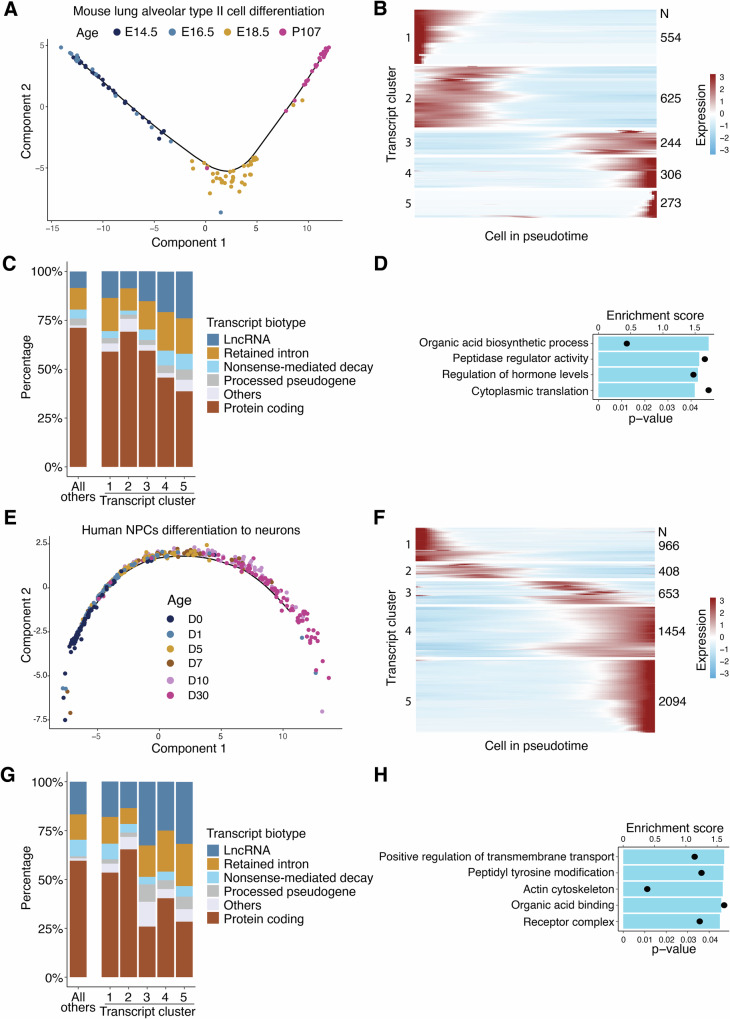
Emergence of non-coding transcript biotypes is a general feature of terminal differentiation. **A**–**D** Analyses of scRNA-seq data on mouse lung alveolar type II (AT2) cell development. **E**–**H** Analyses of scRNA-seq data on in vitro differentiation of human neural precursor cells (NPCs) to neurons. **A**, **E** Monocle-generated trajectory plots representing differentiation pseudotime ordering of mouse AT2 cells (**A**) and human neurons (**E**). E, embryonic day; P, postnatal day; D, day. **B**, **F** Scaled and centered expression heatmaps of transcripts with dynamic expression over the mouse AT2 cells (**B**) and human neurons (**F**) differentiation pseudotimes. Transcripts are ordered by row and cells are by column. Transcripts are clustered by their expression patterns over pseudotime. Number of transcripts per cluster is shown. **C**, **G** Bar charts showing the relative proportions of transcript biotypes in each pseudotime-dependent transcript clusters and in all cells (**C**, mouse AT2 cells; **G**, human neurons). All others: 11,052 all other detected transcripts with TPM > 10 in more than 9 AT2 cells (**C**), and 22,291 all other detected transcripts with TPM > 10 in more than 49 neurons. Chi-squared test: *p* < 2.2 × 10^−16^ for protein-coding vs. non-coding transcript numbers across all clusters, including “All others”. **D**, **H** Top gene ontology terms associated with genes encoding non-coding transcript biotypes in transcript clusters 4 and 5 (highest expression in late/differentiated mouse AT2 cells (**D**) and human neurons (**H**)). Bar magnitude, enrichment score; dot, *p*-value. See also Supplementary Figs. 9–13.

For each cell type, we performed trajectory analysis and generated a pseudotime of cell differentiation based on gene expression using Monocle (mouse AT2: Fig. 4A, human neuron: Fig. 4E, mouse myocyte and mouse neuron: Supplementary Fig. 9A, mouse GP: Supplementary Fig. 10A). As we did for MC, we employed Monocle to identified transcripts with dynamic expression patterns along the differentiation pseudotime. Similar to MC differentiation, the dynamically expressed transcripts clustered into five sequential pseudotime-dependent expression patterns with cluster 5 containing transcripts that emerged in fully differentiated cells (mouse AT2: Fig. 4B, human neuron: Fig. 4F, mouse myocyte: Supplementary Fig. 9B, mouse neuron: Supplementary Fig. 9E, mouse GP: Supplementary Fig. 10B). For each cell type, we then analyzed the relative composition of annotated transcript biotypes for all expressed transcripts and for transcripts in each pseudotime-dependent transcript cluster. Consistent with the observation in MC differentiation, we found that for each cell type, the transcripts in cluster 5 contained a high proportion of non-coding biotypes (mouse AT2: Fig. 4C, human neuron: Fig. 4G, mouse myocyte: Supplementary Fig. 9C, mouse neuron: Supplementary Fig. 9F, mouse GP: Supplementary Fig. 10C). We then performed GO enrichment analysis of genes associated with the non-coding transcripts expressed during cellular differentiation (clusters 4 and 5). We found that the non-coding transcripts which emerged during differentiation were enriched for tissue function-related gene ontologies (mouse AT2: Fig. 4D, human neuron: Fig. 4H, mouse myocyte: Supplementary Fig. 9D, mouse neuron: Supplementary Fig. 9G, mouse GP: Supplementary Fig. 10D). We found these differentiating cells exit the cell cycle very early in differentiation (Supplementary Fig. 11). Across the different cell types there was no consistency in how the expression of splicing factors shifted during differentiation (Supplementary Fig. 12). Taken together, these data suggest that the emergence of non-coding transcripts is a general property of terminal differentiation in mammalian cells and is not solely related to either cell cycle withdrawal or global splicing factor expression changes.

To address the possibility that the emergence of non-coding transcripts during terminal differentiation was somehow an artifact of the Smart-seq sequencing protocol, we further validated this finding by analyzing published long-read single-nuclei full-length RNA sequencing data of in vitro differentiated C2C12 myoblasts to myotubes [33]. Seurat clustering identified three cell clusters (Supplementary Fig. 13A) and Monocle trajectory analysis generated a cell differentiation trajectory (Supplementary Fig. 13B), which agreed with the cell state of myoblast verses myotube (Supplementary Fig. 13C). Expression of proliferation marker *Mki67* was predominantly in the myoblast cell cluster 0 and myocyte differentiation markers *Myog* and *Myh3* were largely in cluster 2 (Supplementary Fig. 13D). Cells in cluster 1 were from the *Pax7* expressing mononucleated subpopulation identified in the original study as being in varying stages of differentiation [33]. These findings confirmed a differentiation trajectory from undifferentiated myoblasts in cluster 0 to differentiated myotubes in cluster 2. We used Monocle to identify transcripts that changed as a function of differentiation and grouped expression patterns into modules (Supplementary Fig. 13E). We classified the transcript modules into three groups (group A, B and C), based on if the highest transcript expression was observed in cell cluster 0, 1, or 2. We analyzed the transcript biotype composition of the three transcript groups and found that transcript group C, which contained modules with transcripts that were upregulated in mature myotubes, was highly enriched with non-coding biotypes, dominated by RI and lncRNA transcripts. Transcript group B showed a similar skewing toward non-coding biotypes among transcripts that emerge during differentiation. In comparison, transcript group A had a lower percentage of non-coding biotypes differentially expressed in undifferentiated myocytes (Supplementary Fig. 13F). These data confirm the emergence of non-coding RNA biotypes as a feature of terminal differentiation using an orthogonal sequencing technology and analysis approach.

### The prominence of non-protein coding RNA biotypes is blunted in terminally differentiated Down syndrome neurons

**Fig. 5:**
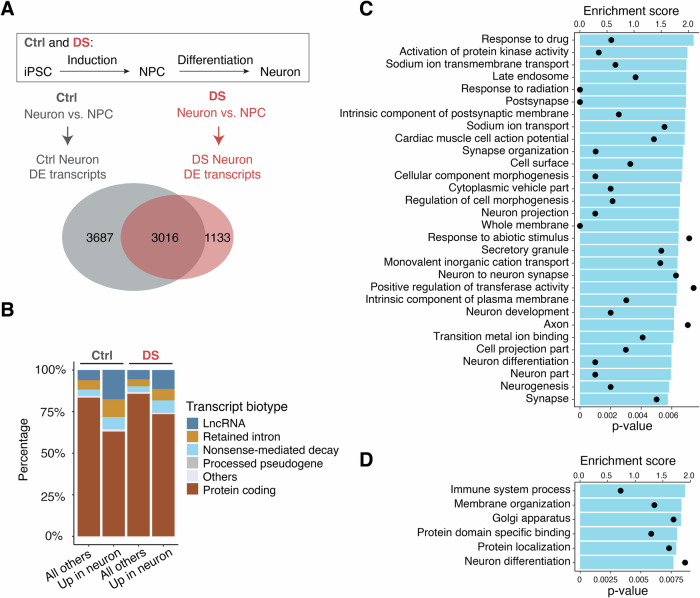
Emergence of non-coding transcript biotypes is diminished in differentiating Down syndrome (DS) neurons. **A** Top: schematic illustration showing the in vitro differentiation of induced pluripotent stem cells (iPSCs) established from healthy control (Ctrl) and Down syndrome (DS) embryos into neural precursor cells (NPCs) and neurons. Middle: schematic illustration showing the identification of differentially expressed (DE) transcripts upregulated in neurons relative to NPCs derived from control (left) and DS (right) iPSCs. Lower: Venn diagram showing the intersection of DE upregulated transcripts identified in control and DS neuronal differentiation. **B** Bar chart showing the relative proportion of transcript biotypes for differentially upregulated and all other transcripts uniquely identified in control or DS neuronal differentiation. All others, expressed transcripts not upregulated in neuron samples (N for Ctrl = 8798; N for DS = 9104). Comparison of protein-coding and non-coding proportions for transcripts upregulated in Ctrl neurons versus DS neurons: *p* = 7.63 × 10^−11^. **C**, **D** Top gene ontology terms associated with genes encoding differentially upregulated non-coding transcripts uniquely identified in control (**C**) or DS (**D**) neuronal differentiation. Bar magnitude, enrichment score; dot, *p*-value. See also Supplementary Fig. 14.

Neurogenesis has been reported to be altered in the brain of Down syndrome (DS) patients [34]. As differentiating neurons demonstrated an upregulation of non-coding transcripts, we examined transcript biotype profiles in neurons differentiated from DS neuronal progenitor cells (NPCs). Sobol et al [35]. established induced pluripotent stem cells (iPSCs) from DS (*n* = 2) and healthy (*n* = 2) human fetal brain, differentiated the iPSCs to NPCs and then to neurons. Using deep bulk RNA-seq, they characterized the transcriptome of the NPCs and neurons from DS and healthy controls. We analyzed the RNA-seq reads from this study at the transcript level and identified differentially expressed (DE) transcripts that were upregulated in neurons compared to NPCs in DS and controls. 3016 transcripts were shared between the DE upregulated transcripts in DS neurons and those in control neurons (Fig. 5A). For the 3687 DE transcripts that were uniquely upregulated in control neurons, 63% were annotated as protein-coding, whereas the 1133 DE transcripts uniquely upregulated in DS neurons had proportionally more (73.5%) protein-coding transcripts (Fig. 5B). GO enrichment analysis revealed that the DE non-coding transcripts uniquely upregulated in control neurons were associated with genes enriched in neuronal differentiation and function (Fig. 5C), while the genes associated with DE non-coding transcripts uniquely upregulated in DS neurons showed less enrichment for neuron differentiation (Fig. 5D). Next, we use the previously analyzed scRNA-seq data from normal human NPCs as they differentiated into neurons and identified a set of 3548 transcripts whose expression were upregulated during terminal differentiation (transcript clusters 4 and 5 of Fig. 4F). When limiting DE transcripts to those present in this neuronal terminal differentiation set, we found a consistent proportional difference, with the transcripts uniquely upregulated in control neurons being 58.9% protein-coding, while DS neurons showed 66.7% coding transcripts (Supplementary Fig. 14). Taken together, these results demonstrate that in differentiated DS neurons, the prominence of non-coding transcripts biotypes – especially those associated with genes important for neuronal function – was decreased. This suggests that decreased emergence of non-coding RNA biotypes and altered neuronal differentiation are part of the impaired neuronal maturation associated with DS.

## Discussion

**Fig. 6:**
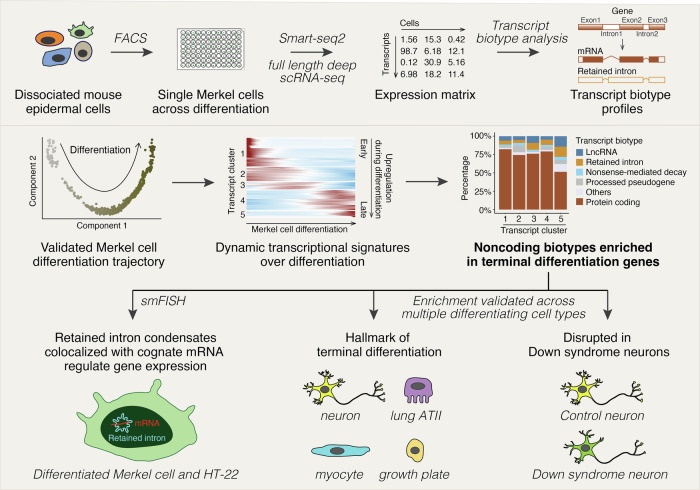
Graphical summary of key study findings. Full-length single cell RNA sequencing identified an enrichment of non-coding transcripts that emerge during the terminal differentiation of Merkel cells. Enriched transcripts included retained intron RNAs capable of forming extrachromosomal nuclear condensates that sequestered cognate mRNAs and decreased protein expression. The emergence of non-coding transcripts was observed during the terminal differentiation of multiple cell types and was blunted in neurons differentiated from patients with Down syndrome.

Applying FACS-based full-length scRNA-seq, we performed deep single-cell sequencing and captured high-quality data to analyze the differentiation of epithelial progenitors to neuroendocrine MCs in mouse skin (Fig. 6). This allowed us to study the developmental trajectory of MCs. By sequencing full-length RNA, we were able to identify transcripts that are dynamically expressed during MC differentiation, including non-coding transcript biotypes such as RI, lncRNA, and NMD. Surprisingly, we found that the proportion of non-coding biotypes was increased among transcripts that emerged during MC terminal differentiation and that these were associated with genes important to MC function. We further showed that RI transcripts found in differentiated MCs formed nuclear condensates that induced nuclear retention of cognate mRNA and downregulated protein expression levels. By analyzing full-length scRNA-seq data of other cell types and using long-read sequencing data, we found that enrichment of non-coding biotypes was a general feature of transcripts that are upregulated during cellular differentiation. This enrichment was diminished in neurons differentiated from patients with DS, suggesting this is a feature of the impaired neuronal maturation seen in DS.

This study has several limitations. First, we dissociated mouse skin and acquired single MCs via cell sorting. While the process required less than three hours and was performed at a low temperature, cell stress still has the potential to introduce transcriptional changes. Despite this limitation, the absence of in vitro models of MC differentiation necessitated our approach, which allowed us to study cellular differentiation in a normal tissue microenvironment. Additionally, most of our study used short-read sequencing of full-length RNA transcripts and this method is inherently more prone to errors in transcript isoform quantification compared to long-read sequencing. However, it is important to note that the high sequence coverage from our use of deep RNA sequencing has been reported to reduce the error rate of transcript quantification [36–38]. Moreover, it is necessary to recognize that our single-cell cDNA library preparation and the public short-read scRNA-seq data we analyzed used oligo(dT) selection, therefore our results are enriched for transcripts with poly-A tails. Further study is needed to fully investigate non-coding RNAs lacking poly-A tails. However, a recent publication reported that circular RNAs increase during vascular cell differentiation and are biomarkers for vascular disease [39], which supports our conclusions. Furthermore, it is reassuring that we observed the same enrichment of non-coding transcripts during differentiation when analyzing long-read single-nuclei RNA sequencing data that was generated with both random hexamer priming and oligo(dT) selection [33]. It is further interesting to note the robustness of the enrichment even though cytoplasmic RNAs are not captured with the single-nuclei sequencing. Despite the heterogeneity of the cell types and data sets in our study, we consistently observed the emergence of non-coding biotypes during terminal differentiation. Although expression of these transcripts was synchronous with terminal differentiation, no single mechanism convincingly explained their emergence and it is likely that multiple mechanisms are involved. To gain insight into the potential general functions of non-coding RNA biotypes, we performed GSEA using standard gene set databases, which are predominantly composed of protein-coding genes and do not distinguish between transcript biotypes. Based on our hypothesis that non-coding transcript biotypes – particularly retained introns and transcripts subject to nonsense-mediated decay (NMD) - may exert regulatory functions on their associated coding transcripts, we conducted the analysis using the genes that produce these non-coding transcripts. The reliance on gene-level annotations, in the absence of transcript-level gene sets, represents a limitation of our approach. Consequently, long non-coding RNAs and other transcript-specific features may be underrepresented in the gene set enrichment analysis. Despite these limitations, our results establish the emergence of non-coding biotypes as a hallmark of terminal differentiation.

We performed a comprehensive analysis of full-length scRNA-seq data to investigate the differentiation of multiple cell types, encompassing embryonic development of MCs and lung alveolar type II cells, postnatal differentiation of the mouse growth plate, in vitro differentiation of MEF into neurons and myocytes, and in vitro differentiation of human neuron precursor cells (NPCs) into neurons. Thus, the dynamic expression of transcripts across the pseudotime-line was a unique range of differentiation events for each cell type. We consistently observed a pronounced enrichment of non-coding transcript biotypes within transcript clusters that were upregulated near the end of the pseudotime-line. This was largely restricted to the final differentiation cluster in cells captured from dissociated tissues, but included the three final transcript clusters when human NPCs were differentiated into neurons and an early transcript cluster in the differentiation of MEF into neurons. The artificial timescales of in vitro differentiation protocols and cellular reprogramming events may have contributed to these differences.

Prior studies have reported on global changes in gene expression that occur during cellular differentiation. In general, undifferentiated pluripotent cells express more genes per cell than committed progenitors or differentiated cells [40]. Furthermore, a range of non-coding RNAs display diverse expression patterns as cells undergo specification in the early stages of embryogenesis [18]. It has also been noted that global translation increases during early differentiation and decreases later in the differentiation of multiple cell types [41]. Similarly, RNA processing and splicing factors are downregulated during the reprograming of mouse fibroblast into cardiomyocytes [15]. Here we discovered that the emergence of non-coding transcript biotypes is a hallmark of cells undergoing terminal differentiation. However, we do not attribute the emergence of non-coding transcripts during terminal differentiation to general changes in RNA processing machinery, as there was no significant shift in the proportion of non-coding biotypes across the global transcriptome during differentiation (see Supplementary Fig. 2C, D). In contrast, our observed enrichment of non-coding biotypes was largely restricted to transcripts that upregulate during late differentiation and were important to specific cellular functions in mature MCs. Moreover, we found the emergence of non-coding transcript biotypes to be a general feature of differentiation among multiple cell types, yet the global shifts in the expression of splicing factors were not consistent across the differentiating cell types (see Supplementary Fig. 12). The mechanisms underlying the production of the multiple non-coding transcripts associated with differentiation will require further investigation.

Given that the levels of certain non-coding RNA transcripts have be shown to be cell-cycle-dependent [18], it is possible that cell cycle withdrawal during terminal differentiation is associated with the production of non-coding transcripts. However, the differentiating cells analyzed in this study exit the cell cycle very early in differentiation (see Supplementary Fig. 11). Therefore, it is unlikely that the emergence of non-coding transcripts in late differentiation is directly associated with cell cycle withdrawal. As non-dividing, differentiated cells do not need to produce structural and housekeeping proteins to generate daughter cells [42], their gene expression regulation may shift towards maintaining levels of functionally relevant proteins. These are the genes where we observed an excess of non-coding transcripts emerging in late differentiation.

The most common non-coding RNA biotypes detected in association with differentiation were RI, lncRNA, and NMD transcripts. All of these have been implicated in regulating gene expression. Modifying transcription and post-transcriptional processing are widely recognized functions of lncRNA [43]. Similarly, NMD transcripts have been reported as a regulator of gene expression in multiple cell types [9–11]. Although RI transcripts have the potential to be translated into peptides, previous studies have demonstrated that intron retention can also modulate the expression of genes crucial to lineage development [7, 8]. In the present study, we discovered a novel mechanism of RI-mediated gene regulation whereby *Aspa* RI nuclear condensates induce nuclear retention of *Aspa* mRNA. Thus, we speculate that, like the *Aspa* RI transcripts, other non-coding RNA transcripts emerging in quiescent differentiated cells may be involved in the regulation of genes important for the function of the mature cells.

The utilization of scRNA-seq enables the investigation of splicing and transcript variants at the individual cell level. Earlier studies detected non-coding transcripts in single cells at a limited scale, primarily due to low gene expression and technical limitations such as low capture efficiency [36, 44]. Our deep and full-length RNAseq on single MCs consistently detected non-coding transcript biotypes allowing us to identify those that were associated with the different stages of cellular differentiation. Although we discovered a novel gene regulatory role for *Aspa* RI transcripts, further studies are warranted to elucidate the precise functions of other non-coding transcripts that emerge during cellular differentiation. Nevertheless, our findings that non-coding biotype expression is consistently enriched as cells terminally differentiate suggest a functional importance for these transcripts. Potential functions include the post-transcriptional regulation of genes required for cell-specific functions. It is also interesting to speculate that dysregulation of this process may take place in cells with defective cellular differentiation, such as neurons of individual with DS.

## Supplementary information

Supplementary Information

Supplementary Video 1

Supplementary Video 2

## Data Availability

The scRNA-seq data from this study is available in the NCBI Gene Expression Omnibus (GEO) under the accession number GSE213104. All analyses were performed in R and Python using previously published packages. No new scripts were created. Scripts employed in this study are available upon request.
