# Supplementary material for: Single cell analysis of developing Merkel cells reveals the emergence of non-coding RNA biotypes as a hallmark of terminal differentiation
Source: Cell Death Differ. 2026 Feb 3;33(7):1503–13. doi: 10.1038/s41418-026-01663-3 (PMC13342250; doi:10.1038/s41418-026-01663-3)
Supplement: Supplementary file 1 — Supplementary Information [file 41418_2026_1663_MOESM1_ESM.pdf]

Supplementary Information for

Single cell analysis of developing Merkel cells reveals the emergence of non-coding RNA biotypes as a hallmark of terminal differentiation

Lingling Miao[1], Loren Collado[1], Savannah Barkdull[1], Patrick Hallaert[1], Mackenzie R. Martin[1], Berkley E. Gryder[2], Michael C. Kelly[3], Stefania Dell'Orso[4], Matthew W. Kelley[3], and Isaac Brownell[1, *]

[1] Dermatology Branch, National Institute of Arthritis and Musculoskeletal and Skin Diseases, National Institutes of Health, Bethesda, MD, USA.
[2] Department of Genetics and Genome Sciences, Case Western Reserve University, Cleveland, OH, USA
[3] Laboratory of Cochlear Development, National Institute on Deafness and Other Communication Disorders, National Institutes of Health, Bethesda, MD, USA
[4] Genomic Technology Section, National Institute of Arthritis and Musculoskeletal and Skin Diseases, National Institutes of Health, Bethesda, MD, USA
Correspondence: isaac.brownell@nih.gov

This pdf file includes:

Materials and Methods

Supplementary Figures 1 to 14

Legends for Supplementary Videos 1 and 2

MATERIALS AND METHODS

Key resources table

REAGENT or RESOURCE	SOURCE	IDENTIFIER
Antibodies		
Rat anti-K8	DSHB	TROMA-I
Rabbit anti-ASPA	ThermoFisher	13244-1-AP
Chemicals, peptides, and recombinant proteins		
Dispase	Corning	354235
Critical commercial assays		
Illumina Nextera XT library preparation kit	Illumina	FC-131-1096
Illumina Nextera XT index kit v2	Illumina	FC-131-2001, 2002, 2003, 2004
RNAScope Multiplex Fluorescent V2 Assay	ACD	323100
Mouse Aspartoacylase (ASPA) ELISA Kit	Mybiosource	MBS7207768
Deposited data		
Raw and analyzed data	This paper	GSE213104
Mouse reference genome: GRCm38.p6	Genome Reference Consortium	https://www.ncbi.nlm.nih.gov/grc/mouse
Human reference genome: GRCh38.p13	Genome Reference Consortium	https://www.ncbi.nlm.nih.gov/grc/human
Reference genome annotation	Ensembl	Release 98, 112
MsigDB (Molecular Signatures Database)	UC San Diego, Broad institute	https://www.gsea-msigdb.org/gsea/msigdb
scRNA-seq: mouse lung alveolar type II cell development	Treutlein *et al.*	GSE52583
scRNA-seq: *in vitro* differentiation of human neural precursor cells to neurons	Wang *et al.*	GSE102066
scRNA-seq: *in vitro* differentiation of mouse embryonic fibroblasts to neurons and myocytes	Treutlein *et al.*	GSE67310
scRNA-seq: mouse growth plate differentiation	Li *et al.*	GSE76157
RNA-seq: Down syndrome iPSCs-derived NPCs and neurons	Sobol *et al.*	N/A

Experimental models: Organisms/strains		
Mouse: *Sox2*$^{GFP/+}$	JAX	017592
Mouse: *Gfi1*$^{GFP/+}$	JAX	016162
Oligonucleotides		
FISH probe targeting sequence: *Aspa* mRNA probe #1, 2-631bp of ENSMUST00000021119	ACD	NA
FISH probe targeting sequence: *Aspa* mRNA probe #2, 658-1486bp of ENSMUST00000021119	ACD	NA
FISH probe targeting sequence: *Aspa* RI, 1684-3435bp of ENSMUSE00000766546	ACD	NA
FISH probe targeting sequence: *Vwa5b2* mRNA, 3240-4169bp of NM_001144953.1	ACD	NA
FISH probe targeting sequence: *Vwa5b2* RI, 20-776bp of ENSMUST00000145483.1	ACD	NA
Software and algorithms		
Bcl2fastq2	Illumina	v2.20
Trimmomatic	https://github.com/usadellab/Trimmomatic	v0.36
STAR	https://github.com/alexdobin/STAR	v2.5.2b; v2.7.10b
RSEM	https://github.com/deweylab/RSEM	v1.3.0; v1.3.3
Seurat	https://satijalab.org/seurat/	v2.3.0; v4.3.0
Monocle	http://cole-trapnell-lab.github.io/monocle-release/	Monocle 2; Monocle 3
RSeQC	https://rseqc.sourceforge.net/	V3.0.1
R	https://www.r-project.org/	v3.5.0 - v4.2.2
DESeq2 R package	https://bioconductor.org/packages/release/bioc/html/DESeq2.html	v1.26.0; v1.36.0
ggplot2 R package	https://cran.r-project.org/web/packages/ggplot2/index.html	v3.0.0 – v3.3.6

ImageJ	https://imagej.net/ij/index.html	v1.52q
Imaris	https://imaris.oxinst.com/	10.2.0

Method details

Mouse

Sox2$^{GFP/+}$ and *Gfi1*$^{GFP/+}$ reporter mice were purchased from JAX and were housed and bred on an outcrossed Swiss Webster background in the animal facility at the National Cancer Institute and the National Institute of Arthritis and Musculoskeletal and Skin Diseases, NIH. All experiments were performed in accordance with institutional guidelines and IACUC-approved protocols. Mice of different ages were collected to harvest skin samples for cell sorting and single-cell analysis. Sample sizes were based on previous experience and availability, and no formal statistical sample size calculation was performed. For scRNA-seq, investigators were aware of the genotype at the time of skin collection; however, all downstream analyses, including cell sorting, sequencing and bioinformatic processing, were performed using coded sample identifiers to ensure blinding. For cell sorting, all pups of the specified genotype were included, and no further randomization was applied. For smFISH experiments, mice were randomly selected from the available wild-type littermates.

Epidermal cell dissociation

Neonatal mice (postnatal day 0, P0) were euthanized in a CO_2 euthanasia chamber for 20 minutes, followed by decapitation and skin harvesting. To dissociate epidermal cells, neonatal skin was incubated in dispase (Corning, #354235) for 1 hour at room temperature. The epidermal sheet was then separated from the dermis. The epidermis

was subsequently cut into small pieces and digested in 0.05% trypsin for 15 minutes, with shaking occurring every 5 minutes. DMEM with 10% FBS was added to stop enzyme digestion. The epidermal suspension was pipetted up and down ten or more times, passed through a 70 µM and then a 40 µM cell strainer, and was then collected in a new Falcon tube. Epidermal cells were pelleted by centrifuge at 300 x g for 5 minutes. The resulting supernatant was carefully aspirated without disturbing the cell pellet. The cell pellets were then resuspended in PBS with 4% FBS.

Postnatal day 6 (P6) mice were euthanized following same protocol as for P0 mice. Skin harvested from P6 mice was incubated in 0.15% trypsin at 37°C for 30 minutes by floating the skin dermis-side down on top of the trypsin. The epidermis was subsequently collected by scraping with a scalpel in 0.05% trypsin. Postnatal epidermal cells were then processed by following the same procedure used for dissociated neonatal epidermis.

To prepare embryonic mouse skin cells, female breeders were euthanized in a CO_2 euthanasia chamber for 3 minutes followed by cervical dislocation. Embryos at days (E) 16.5, 17.5 or 18.5 were then harvested and euthanized by decapitation. Embryonic skin was harvested and processed using the same procedure as described for neonatal skin, with (E18.5) or without (E16.5 and E17.5) dispase treatment.

For each time point and each mouse strain ($Sox2^{GFP/+}$ or $Gfi1^{GFP/+}$), single cells sorted from one litter (3-5 GFP-positive mice) were processed for cDNA library preparation and included in the pooled library for sequencing, except that P0 $Sox2^{GFP/+}$ cells were collected from 8 mice across 3 litters.

Flow cytometry and cell sorting

Neonatal or postnatal epidermal and embryonic skin cells from *Sox2*$^{GFP/+}$ or *Gfi1*$^{GFP/+}$ reporter mice were stained for viability with DAPI and analyzed by flow cytometry on a BD LSRII analyzer. Single $GFP^{+}DAPI^{-}$ cells were sorted into a 96-well plate preloaded with lysis buffer using Beckman MoFlo Astrios or BD Influx Cell Sorter. This lysis buffer was prepared according to the Smart-seq2 protocol (1). Single $GFP^{-}DAPI^{-}$ cells from the neonatal epidermal cells were sorted as controls.

scRNA-seq

Single-cell cDNA libraries were prepared by following the Smart-seq2 protocol (1) with minor modifications. Briefly, single-cell RNA transcripts were reverse-transcribed using SuperScript II Reverse Transcriptase (ThermoFisher, #18064014) followed by 21 cycles of pre-amplification. cDNA concentrations were assessed using the PicoGreen dsDNA Assay kit (ThermoFisher, #P11496), and cDNA quality was evaluated using the Agilent bioanalyzer with the High Sensitivity DNA kit (Agilent, #5067-4626). High quality single-cell cDNA strands were selected for further processing. The cDNA was diluted, and its concentration was normalized. A total of 1152 single-cell cDNA libraries were generated using the Illumina Nextera XT library preparation kit (Illumina, #FC-131-1096) and indexed through the Illumina Nextera XT index kit v2 (Illumina, #FC-131-2001, 2002, 2003, 2004). cDNA libraries from each of the 384 cells were pooled together for sequencing, inclusion of single cells into libraries was stratified such that each pooled library contained cells sourced all different time points and both reporter mice. This resulted in a total of three pooled cDNA libraries being prepared. Each pooled library was paired-end sequenced across 4 lanes of the Hiseq3000 with 125bp or 100bp read length.

scRNA-seq data processing

Sequencing reads were demultiplexed using Bcl2fastq v2.20. Adapters were trimmed, as were 125bp sequencing reads which were trimmed to 100bp using Trimmomatic v0.36. Reads were aligned to the GRCm38.p6 mouse reference genome using STAR v2.5.2b with Ensembl annotation (release 98). Expression values for individual genes and transcripts were both estimated and quantified with RSEM v1.3.0 and TPM (transcripts per million) values were used for further analysis. Index hopping correction was applied, and all subsequent analyses were performed on the corrected data (2).

scRNA-seq data analysis

Out of 1057 cells that passed quality control (defined as having more than 2000 expressed genes and less than 10% mitochondrial gene expression), 698 single cells were identified as MCs and MC precursors. TPM gene or transcript expression matrices were used, and analyses were conducted using default parameters unless otherwise specified.

The trajectory of MC differentiation was analyzed using Monocle 2 (3). Differentially expressed genes were identified in an unsupervised manner, and single cells were ordered based on expression of top 1905 differentially expressed genes (adjusted p-value < 0.00001). For cell ordering, dimensionality reduction was performed using the *DDRtree* method. Pseudotime-dependent genes were then identified.

Transcripts differentially expressed across MC differentiation pseudotime were identified and clustered on their expression patterns using Monocle 2. Biotypes for individual transcripts were annotated using Ensembl BioMart. The biotype composition for each transcript cluster was analyzed. Genes expressing non-coding transcripts that

increased during terminal differentiation were analyzed for gene ontology using Gene Set Enrichment Analysis.

Identification of detected transcripts and analysis of transcript biotype composition

To determine which transcripts were reliably detected in single MCs, we counted the number of single cells with an expression level of TPM > 10 for each transcript. The ROSE (Rank Ordering of Super-Enhancers) method was used to plot the rank ordering of cell numbers for all transcripts, and a threshold of > 58 cells was identified. Thus, we determined that transcripts with TPM > 10 in more than 58 single MCs were reliably detected in our scRNA-seq data. Transcripts not included in the pseudotime-dependent transcript clusters/modules were categorized as “All others” (N=13600). Using the same method, we determined that 11052 all other transcripts (TPM > 10 in more than 9 cells) were detected in the mouse AT2 data (4), 22291 all other transcripts (TPM > 10 in more than 49 cells) were detected in the human neuron data (5), 13004 all other transcripts (TPM > 10 in more than 21 cells) and 16308 all other transcripts (TPM > 10 in more than 14 cells) were respectively detected in the mouse MEF/myocyte and MEF/neuron data (6), 21183 all other transcripts (TPM > 10 in more than 7 cells) were detected in the mouse growth plate data (7), and 20562 all other transcripts (TPM > 10 in more than 1 cell) were detected in the long-read single-nuclei RNA-seq data on myotube differentiation (8). Biotypes of individual transcripts were annotated using Ensembl BioMart. The proportion of each biotype was then calculated for transcripts within each pseudotime-dependent transcript cluster and for all other detected transcripts. The most representative transcript biotypes detected were protein coding, RI, lncRNA, NMD, and

processed pseudogene, whereas the remaining biotypes were assigned to the category "others". Pearson's chi-squared tests were conducted to assess the distribution of protein coding and non-coding transcripts among all transcript clusters, including the "All others" category.

Analysis of published datasets

We selected full-length short-read scRNA-seq data studying cell differentiation that includes paired-end sequencing generated using Smart-seq as well as read lengths no shorter than 100 bp. Our selected datasets include: mouse lung alveolar type II cell development ((4), GEO accession number GSE52583), *in vitro* differentiation of human neural precursor cells (NPCs) to neurons ((5), GEO accession number GSE102066), *in vitro* differentiation of mouse embryonic fibroblasts (MEFs) to neurons and myocytes ((6), GEO accession number GSE67310), and mouse growth plate differentiation ((7), GEO accession number GSE76157). Once FASTQ data were downloaded, data processing and analysis was performed using the same pipeline and tools employed for our MC scRNA-seq analysis. Sequencing reads were aligned to either the GRCm38.p6 mouse reference genome or the GRCh38.p13 human reference genome. All single cells from the datasets were included in the clustering and differentiation trajectory analysis, except for mouse lung alveolar type II cell, in which only the *Sftpc*+ AT2 cell lineage was included. We confirmed that cell clustering and differentiation trajectory results were consistent with those reported in the source literature. For mouse MEFs' differentiation to myocytes and neurons, differentiation was induced either by exclusively overexpressing the proneural pioneer factor *Ascl1* or by simultaneously overexpressing the *Ascl1*, *Brn2*, and *Myt1l* transcription factors (TFs). Differentiation was initiated in

naïve MEFs or clonal *Ascl1*-inducible MEFs. In Supplemental Fig. 9, differentiating cell samples derived from MEFs induced by either the three TFs or clonal *Ascl1* were respectively labeled with a differentiation date followed by "3TFs" or "clonal" to distinguish them from naïve MEF-derived *Ascl1*-induced cells.

Gene and transcript expression matrices from long-read sequencing of C2C12 myoblasts differentiating into myotubes ((8), GSM5169183 from GSE168776) were downloaded, and only single-nuclei data were included for further analysis. Seurat 4.3.0 was used for cell clustering and Monocle 3 was used for trajectory analysis using the gene expression matrix. Transcript modules that changed as a function of differentiation pseudotime were also identified using Monocle 3. These modules were then classified based on their expression patterns, specifically the highest levels in different cell clusters. Biotypes of individual transcripts were annotated using Ensembl BioMart, and biotype composition for transcripts in each group was analyzed.

FASTQ RNA-seq data on iPSCs-derived NPCs and neurons from healthy controls and DS patients were kindly provided by Dr. Niklas Dahl's research group (9). Sequencing reads were mapped to the GRCh38.p13 human reference genome using STAR v2.5.2b with Ensembl annotation (release 98). Expression at both the gene and transcript levels was estimated and quantified with RSEM v1.3.0. For both healthy control and DS conditions, differentially expressed transcripts between NPCs (n=2) and differentiated neurons (n=2) were identified using DESeq2 v1.26.0. The biotypes for individual transcripts were annotated using Ensembl BioMart. For both healthy control and DS conditions, expressed transcripts were identified by filtering those with TPM greater than 10 in more than 2 samples, ensuring their expression in both NPCs and

neurons. Expressed transcripts that were not differentially upregulated during neuronal differentiation were categorized as “All others” (N=8798 for healthy control and N= 9104 for DS). The transcript biotype composition was analyzed for all other detected transcripts as well as for those upregulated in differentiated neurons. A two-proportion z-test with continuity correction was conducted to assess the equality of proportions for protein coding and non-coding transcripts upregulated in healthy control neurons versus DS neurons.

smFISH

Neonatal mice were euthanized in a CO_2 euthanasia chamber for 20 minutes followed by decapitation. After harvesting their dorsal skin, the epidermal sheet was separated from the dermis using dispase (Corning, #354235). This epidermal sheet was then fixed in 4% PFA at 4°C overnight. Whole mount smFISH was performed on the epidermal sheet using the RNAScope Multiplex Fluorescent V2 Assay (ACD, #323100) following the manufacturer’s instructions. Isoform-specific smFISH probe targets included: *Aspa* mRNA probe #1, 2-631bp of ENSMUST00000021119; *Aspa* mRNA probe #2, 658-1486bp of ENSMUST00000021119; *Aspa* RI probe, 1684-3435bp of ENSMUSE00000766546; *Vwa5b2* mRNA probe, 3240-4169bp of NM_001144953.1; and *Vwa5b2* RI probe, 20-776bp of ENSMUST00000145483.1. Following smFISH, K8 (DSHB, TROMA-I) immunostaining was performed on the epidermis to visualize MCs. Confocal images were acquired using a Leica SP8 LIGHTNING confocal system. For each touch dome, MCs were equally assigned to the inner and outer touch dome based on their location. smFISH of HT-22 cells cultured on coverslips was conducted and analyzed using the same methods. Nuclear signals were counted in the DAPI stained

area. For *Aspa* mRNA, mRNA probe #1 was used for MCs and mRNA probe #2 was used for HT-22 cells.

Signal intensity for all RNA targets was analyzed using ImageJ or Imaris software. For *Aspa* and *Vwa5b2* transcripts in MCs, signal intensity for each individual transcript was measured in each cell within a touch dome and then normalized to the mean intensity per cell for that specific touch dome. The relative intensity in single cells within the inner and outer touch domes was shown in Fig. 2C (*Aspa*) and Supplementary Fig. 5F (*Vwa5b2*). Colocalization of coding and non-coding transcripts was assessed for each cell using Coloc 2 function in ImageJ, and data for individual cells were presented in Supplementary Fig. 3D-F (*Aspa*) and Supplementary Fig. 5G-I (*Vwa5b2*). The nuclear and cytoplasmic proportions of *Aspa* mRNA and *Aspa* RI in mouse touch dome MCs were analyzed on z-stacked images in Imaris using the Surpass tool. Signal intensity was analyzed for each MC (indicated by K8 staining) and for their nuclei (segmented by DAPI staining). The proportion of signal in the nuclei versus the whole cell for individual MCs (N =17) was presented in Supplementary Fig. 4A. For *Aspa* transcripts in HT-22 cells, the overall signal intensity within a high-power field (covering 6-28 cells) was collected for each transcript. This intensity was divided by the number of cells in each field to obtain the mean signal intensity per cell. Values from 6 fields for each condition were collected. The mean signal intensity per cell was then normalized relative to the mean value from control groups (undifferentiated cells or vector-transfected cells). The relative signal intensity per cell across different fields was summarized and presented as box plots in Fig. 3A’ and B’. For each *Aspa* transcript, signal intensity was also measured for individual HT-22 cells. The percentage of

cytoplasmic signal was calculated by subtracting the nuclear intensity (marked by DAPI staining) from the whole cell intensity and then dividing by the whole cell intensity. The percentage of cytoplasmic signal for individual cells was shown in Fig. 3A'' and B''. Statistical comparisons of signal intensities or colocalization between the inner and outer touch domes, as well as between two treatments in HT-22 cells, were performed using two-sample t-test, with Welch's correction applied when variances were unequal.

Image files from different smFISH experiments were coded and randomized prior to analysis so that the investigator performing the quantification was blinded to the probe identity.

HT-22 culture, differentiation and transfection

HT-22 cells were generously provided by Dr. Klaus van Leyen at Massachusetts General Hospital, Harvard University. Authentication was performed by short tandem repeat (STR) profiling to confirm identity. Mycoplasma contamination was detected and successfully eliminated using Plasmocin (InvivoGen) at 4.5 µg/ml prior to experimental use. The cells were cultured in DMEM with 10% FBS, 100U/ml penicillin, and 0.1 mg/mL streptomycin. To ensure neuronal differentiation, cells were seeded on coverslips or dishes coated with 100 µg/ml PDL (MilliporeSigma, #P0899) and 10 µg/ml laminin (ThermoFisher, #23017015). The cells were cultured overnight, and the media was then replaced with Neurobasal medium (ThermoFisher, #21103049) supplemented with 2mM L-glutamine (ThermoFisher, #25030081) and 1 X N-2 supplement (ThermoFisher, #17502048) for 48 or 96 hours (96 hours for ELISA, 48 hours for all other experiments) before harvesting (10). Differentiation was assessed using cell cycle

analysis, cell morphology assay and RT-qPCR quantification for the expression of *Chat*, which is known to be upregulated in HT-22 cells upon neuronal differentiation (11, 12).

DNA encoding the full-length mouse *Aspa*-202 transcript was synthesized by Biomatik and subsequently cloned into a pcDNA3.1 vector. The overexpressing plasmid or empty vector control were then transfected into undifferentiated HT-22 cells using Lipofectamine 2000 (ThermoFisher, #11668019). Cells were harvested 24-48 hours post-transfection.

siRNA targeting mouse *Aspa* and negative control were purchased from ThermoFisher. NC: Catalog No. 4390844; siRNA#1: siRNA ID s61939; siRNA#2: siRNA ID s61940; siRNA#3: siRNA ID s61941. siRNAs were transfected into undifferentiated HT-22 cells using Lipofectamine 3000 (ThermoFisher, #L3000008). Cells were harvested 48 hours post-transfection for RT-qPCR analysis of *Aspa* mRNA expression.

To detect the function of ASPA or *Aspa* RI on HT-22 cell neuronal differentiation, HT-22 cells were transfected with *Aspa*-202 overexpressing plasmid or empty vector control, or *Aspa* siRNAs or negative control, and changed to differentiation media 48 hours post-transfection. Cells were harvested for analysis 48 hours post-differentiation.

ASPA flow cytometry and ELISA

HT-22 cells were harvested, stained with the LIVE/DEAD Fixable Violet Dead Cell Stain Kit (ThermoFisher, #L34963), and fixed in 4% PFA for 30 minutes. The cells were then permeabilized with 0.2% Tween 20 at room temperature for 30 minutes. Subsequently, the cells were stained with APC-conjugated anti-ASPA antibody (APC conjugation kit: Abcam, #ab201807; ASPA antibody: ThermoFisher, # 13244-1-AP) for 90 minutes at room temperature. These stained cells were analyzed using an Attune flow cytometer

(ThermoFisher). The resulting data were processed in FlowJo such that representative histograms were normalized to unit area to ensure comparability of ASPA expression levels across samples. Mean fluorescence intensity (MFI) was retrieved for statistical analysis, and the two treatments in HT-22 cells were compared via a two-tailed unpaired Student's t-test.

ASPA ELISA was conducted to assess ASPA protein expression using a mouse ASPA ELISA kit (Mybiosource, #MBS7207768) following manufacturer's instructions. Briefly, undifferentiated HT-22 cells transfected with the *Aspa*-202 overexpressing plasmid or empty vector, as well as undifferentiated and differentiated HT-22 cells were harvested and lysed by performing three cycles of freeze-thaw. Cell lysates were collected after centrifugation to remove debris and proceed with the assay. Experiments were conducted in triplicates. ASPA protein concentration was normalized to total protein quantified by BCA assay (ThermoFisher, #23227), and relative protein expression was calculated by normalizing to the control (empty vector for *Aspa*-202 overexpression and undifferentiated cells for differentiation). Data were compared using a two-tailed unpaired Student's t-test.

RNA extraction, RT and qPCR

RNA was extracted using the RNeasy Mini Kit (Qiagen cat. #74134) and cDNA was synthesized with the Superscript III First-Strand Synthesis System (ThermoFisher Scientific, cat. #18080051) according to manufacturer's instructions. cDNA was synthesized using random hexamers. qPCR for *Aspa* and *Chat* was performed with power SYBR Green PCR master mix (Thermofisher, #4368702) and analyzed on StepOnePlus real-timePCR system (Thermofisher). The relative *Aspa* and *Chat* mRNA

levels were analyzed by normalizing the threshold cycle (Ct) value to that of internal loading control, *Rpl13a*. Primers used are: *Aspa* forward 5'-CCTCACGGTGTCCTTAGAGC -3'; *Aspa* reverse 5'-ATGGTTTCCAGTCTTGATCCTGC -3'; *Chat* forward 5'-GCTTGAATGGAGCGAATCGTTGG -3'; Chat reverse 5'-CACCAGGACGATGCCATCAAAAG -3'; Rpl13a forward 5'-CTGCTCTCAAGGTTGTTCGGCT -3'; Rpl13a reverse 5'-CCTTCCGTTTCTCCTCCAGAGT -3'. Expression of *Aspa* or *Chat* between different groups (N = 3) was compared using one-way ANOVA.

Cell proliferation, cell cycle, and apoptosis assay, and Giemsa staining

HT-22 cells transfected with the *Aspa*-202 overexpressing plasmid or empty vector were harvested 48 hours post-transfection. Cell numbers were counted using an automated cell counter (DeNovix) and expressed relative to the initial numbers seeded. Cell numbers between the *Aspa* overexpressing and control groups (N = 5) were compared using a two-tailed unpaired Student's t-test. Cell apoptosis was analyzed by flow cytometry. Briefly, harvested cells were washed with PBS and resuspended in Annexin V Binding Buffer (Biolegend, #422201). Cells were then stained with APC-conjugated Annexin V (Biolegend, #640941) and propidium iodide (PI, MilliporeSigma, #P4864) for 15 minutes at room temperature in the dark, followed by analysis on an Attune flow cytometer (ThermoFisher). Apoptosis between *Aspa* overexpressing and control groups (N = 4) was compared using two-way ANOVA.

HT-22 cells transfected with the *Aspa*-202 overexpressing plasmid or empty vector, or *Aspa* or negative control siRNAs, followed by neuronal differentiation and

undifferentiation control cells, were harvested for cell cycle analysis. Briefly, cells were fixed in cold 70% ethanol at -20 °C overnight, then washed with PBS and stained with PI solution (containing 50 µg/ml PI, 100 µg/ml DNase-free RNase A, and 0.05% Triton X-100) at 37 °C for 15 minutes. Stained cells were washed with PBS and analyzed on an Attune flow cytometer (ThermoFisher). Additionally, a Giemsa assay was also conducted on cells with the same transfection and differentiation treatments using the Differential Quik III staining kit (Polysciences, #26419) to assess cell morphology.

RNA-seq of HT-22 cells

HT-22 cells transfected with the *Aspa*-202 overexpressing plasmid or empty vector in triplicates were harvested 48 hours post-transfection. RNA was extracted using the RNeasy Mini Kit (Qiagen cat. #74134). Library preparation was performed using the NEBNext Ultra II RNA Library Prep Kit for Illumina (NEB, #E7770). Sequencing was carried out on a NovaSeq platform using pair-end 50-bp reads. Reads were aligned to the GRCm39 mouse reference genome using STAR v2.7.10b with Ensembl annotation (release 112). Expression values for individual genes were estimated and quantified with RSEM v1.3.3. Differentially expressed genes were identified with DESeq2 v1.36.0.

Statistical analysis

All quantitative methods and statistical analyses are described in the relevant sections of the Method details. Normality of the data was assessed using the Shapiro-Wilk test, and homogeneity of variances was evaluated using Levene's test. For comparisons involving two groups, a standard unpaired t-test was used when variances were equal; otherwise, Welch's t-test was applied. For analyses involving more than two groups or factorial designs, ANOVA was conducted. When variances were equal, standard one-

way or two-way ANOVA was used; in cases of unequal variances, Welch's ANOVA was performed. Statistical analyses were carried out using R or GraphPad Prism.

Supplementary References:

1. Picelli S, Faridani OR, Bjorklund AK, Winberg G, Sagasser S, Sandberg R. Full-length RNA-seq from single cells using Smart-seq2. Nat Protoc. 2014;9(1):171-81.
2. Miao L, Collado L, Barkdull S, Saito Y, Jo JH, Han J, et al. Estimating and correcting index hopping misassignments in single-cell RNA-seq data. bioRxiv. 2024.
3. Trapnell C, Cacchiarelli D, Grimsby J, Pokharel P, Li S, Morse M, et al. The dynamics and regulators of cell fate decisions are revealed by pseudotemporal ordering of single cells. Nat Biotechnol. 2014;32(4):381-6.
4. Treutlein B, Brownfield DG, Wu AR, Neff NF, Mantalas GL, Espinoza FH, et al. Reconstructing lineage hierarchies of the distal lung epithelium using single-cell RNA-seq. Nature. 2014;509(7500):371-5.
5. Wang J, Jenjaroenpun P, Bhinge A, Angarica VE, Del Sol A, Nookaew I, et al. Single-cell gene expression analysis reveals regulators of distinct cell subpopulations among developing human neurons. Genome Res. 2017;27(11):1783-94.
6. Treutlein B, Lee QY, Camp JG, Mall M, Koh W, Shariati SA, et al. Dissecting direct reprogramming from fibroblast to neuron using single-cell RNA-seq. Nature. 2016;534(7607):391-5.
7. Li J, Luo H, Wang R, Lang J, Zhu S, Zhang Z, et al. Systematic Reconstruction of Molecular Cascades Regulating GP Development Using Single-Cell RNA-Seq. Cell Rep. 2016;15(7):1467-80.
8. Rebboah E, Reese F, Williams K, Balderrama-Gutierrez G, McGill C, Trout D, et al. Mapping and modeling the genomic basis of differential RNA isoform expression at single-cell resolution with LR-Split-seq. Genome Biol. 2021;22(1):286.
9. Sobol M, Klar J, Laan L, Shahsavani M, Schuster J, Anneren G, et al. Transcriptome and Proteome Profiling of Neural Induced Pluripotent Stem Cells from Individuals with Down Syndrome Disclose Dynamic Dysregulations of Key Pathways and Cellular Functions. Mol Neurobiol. 2019;56(10):7113-27.
10. He M, Liu J, Cheng S, Xing Y, Suo WZ. Differentiation renders susceptibility to excitotoxicity in HT22 neurons. Neural Regen Res. 2013;8(14):1297-306.
11. Lim J, Bang Y, Kim KM, Choi HJ. Differentiated HT22 cells as a novel model for in vitro screening of serotonin reuptake inhibitors. Front Pharmacol. 2022;13:1062650.
12. Liu J, Li L, Suo WZ. HT22 hippocampal neuronal cell line possesses functional cholinergic properties. Life Sci. 2009;84(9-10):267-71.

SUPPLEMENTARY FIGURES 1 to 14

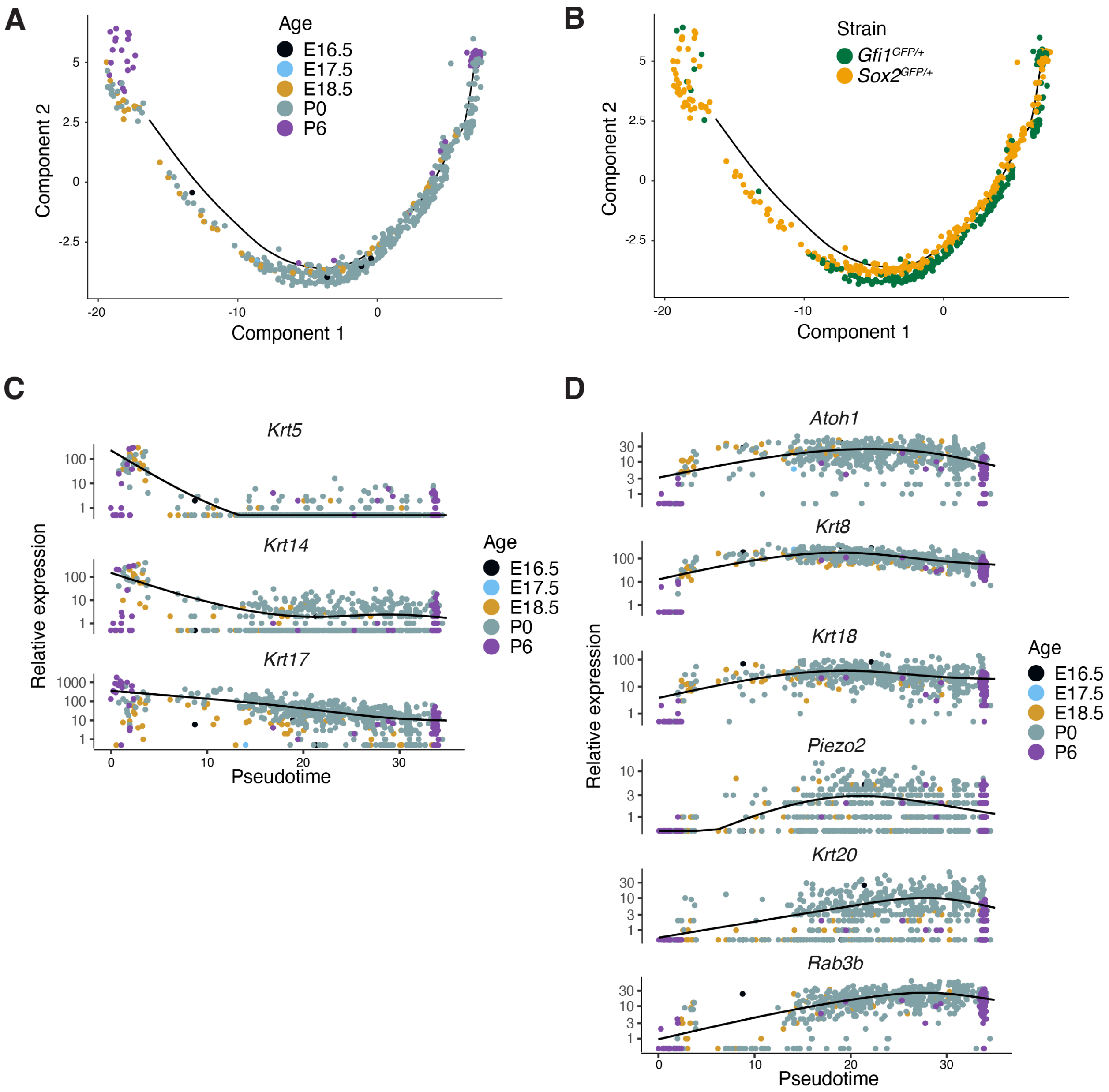

Supplementary Fig. 1 MC marker genes are expressed in differentiating MCs.

(A and B) Same pseudotime plots as Figure 1A labeled with sampling timepoints (A) and source GFP reporter mice (B).

(C and D) Scatter plots indicating the relative expression levels of MC progenitor marker genes (C) and differentiated MC marker genes (D) in single MCs along the pseudotime of MC differentiation. E, embryonic day; P, postnatal day.

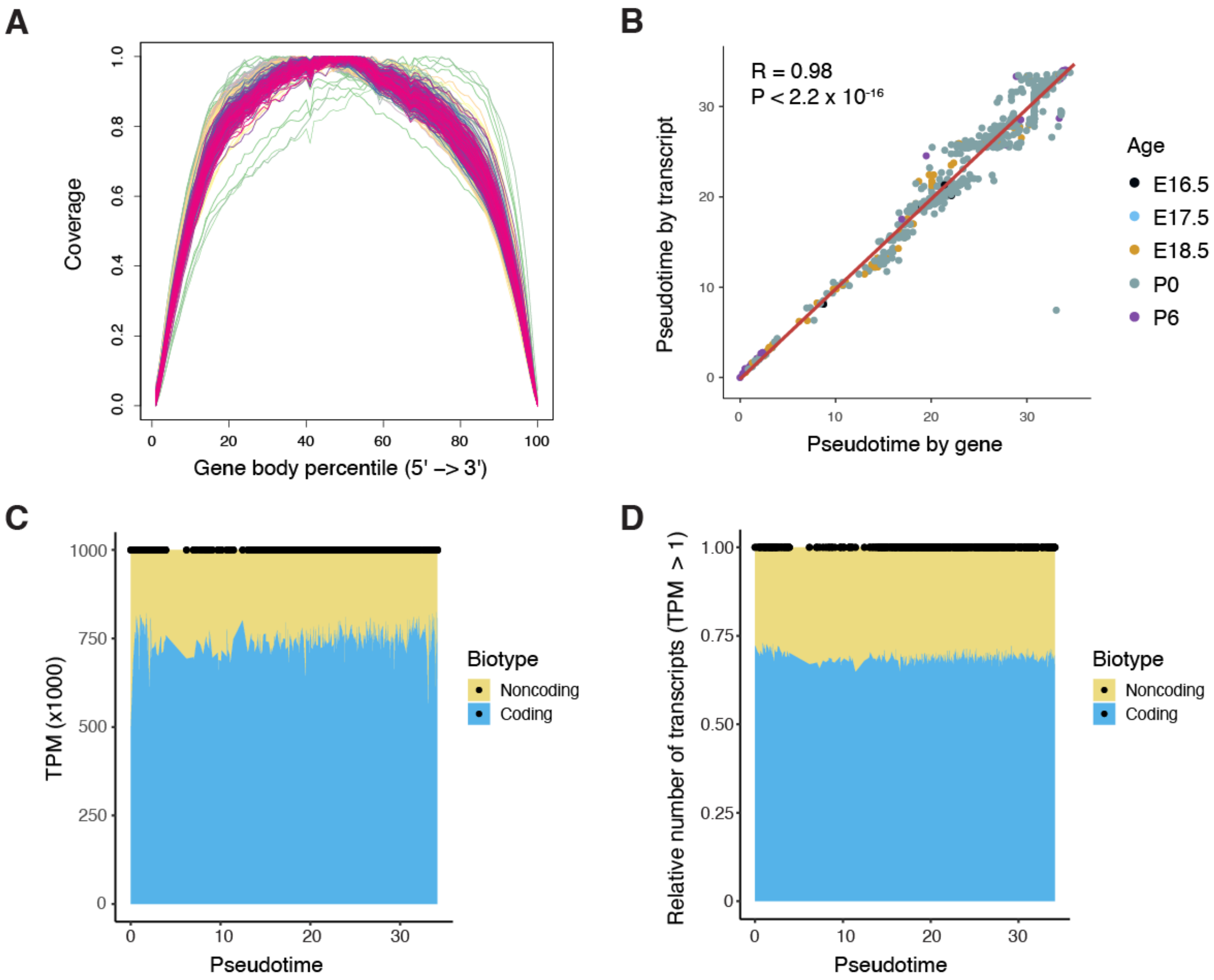

Supplementary Fig. 2 Full-length scRNA-seq captures transcript expression during MC differentiation.

(A) Gene body coverage plot showing read coverage over gene bodies in single MCs. The mean coverage is estimated from housekeeping genes. Each line shows the distribution of reads along the length of the genes from the 5'-end to the 3'-end in a single cell.

(B) Scatter plot showing the correlation of MC differentiation pseudotime estimated using gene expression and transcript expression data. Each dot represents a cell.

(C) Area plot showing the relative expression contribution of protein coding and non-coding transcript reads in single MCs along the pseudotime of differentiation. Total TPM of coding and non-coding transcripts is indicated by the blue and yellow areas respectively. Each dot represents a cell.

(D) Area plot showing the relative number of expressed protein coding and non-coding transcripts with a TPM > 1 in single MCs along the pseudotime of differentiation. Number of coding and non-coding transcripts is indicated by the blue and yellow areas respectively. Each dot represents a cell.

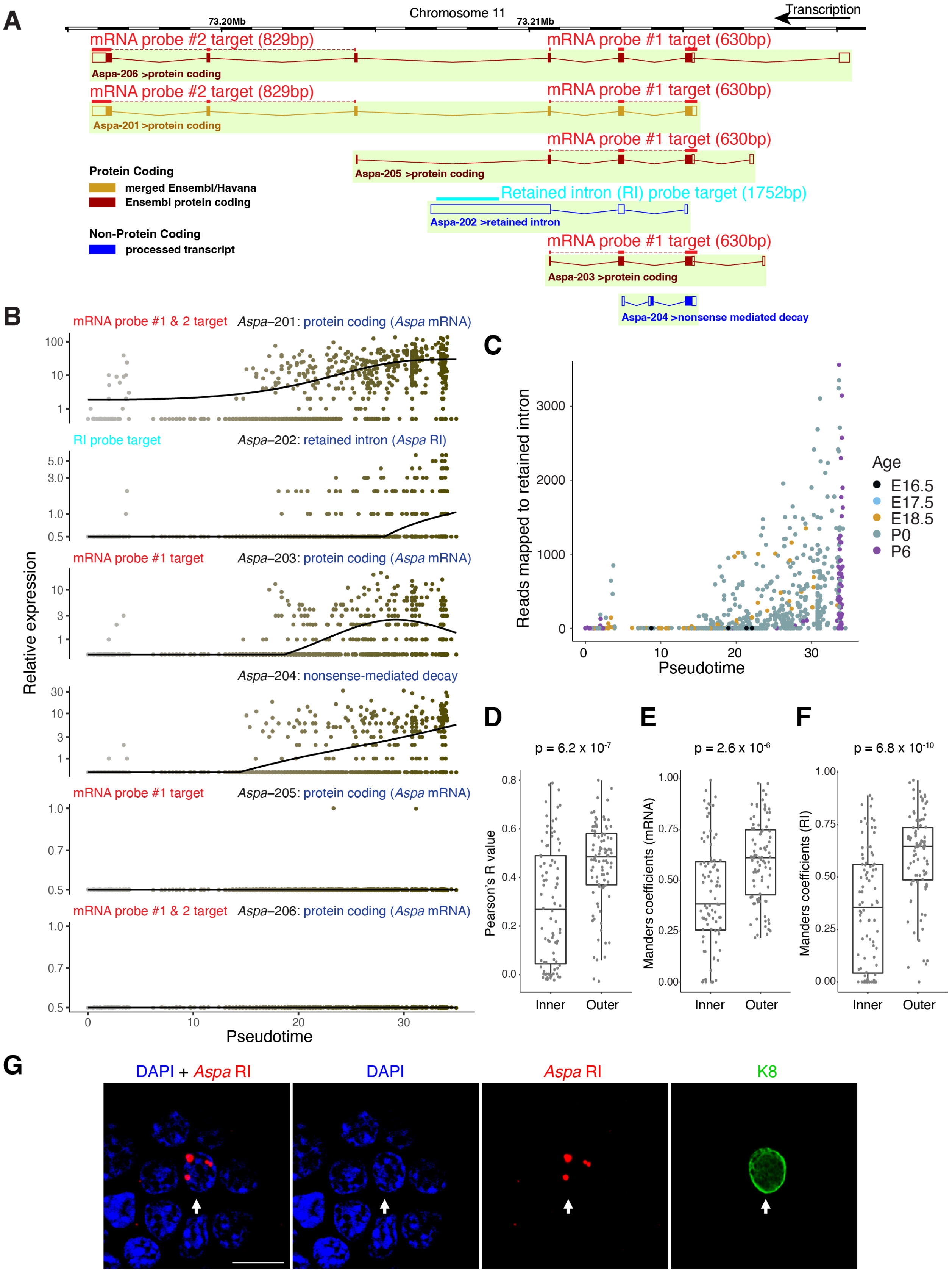
A
Chromosome 11
73.20Mb
73.21Mb
Transcription
mRNA probe #2 target (829bp)
mRNA probe #1 target (630bp)
Aspa-206 >protein coding
Aspa-201 >protein coding
Aspa-205 >protein coding
Retained intron (RI) probe target (1752bp)
Aspa-202 >retained intron
Aspa-203 >protein coding
Aspa-204 >nonsense mediated decay
Protein Coding
merged Ensembl/Havana
Ensembl protein coding
Non-Protein Coding
processed transcript
B
mRNA probe #1 & 2 target
Aspa–201: protein coding (Aspa mRNA)
RI probe target
Aspa–202: retained intron (Aspa RI)
mRNA probe #1 target
Aspa–203: protein coding (Aspa mRNA)
Aspa–204: nonsense-mediated decay
Aspa–205: protein coding (Aspa mRNA)
Aspa–206: protein coding (Aspa mRNA)
Relative expression
Pseudotime
C
Reads mapped to retained intron
Pseudotime
Age
E16.5
E17.5
E18.5
P0
P6
D
p = 6.2 x 10-7
Pearson's R value
E
p = 2.6 x 10-6
Manders coefficients (mRNA)
F
p = 6.8 x 10-10
Manders coefficients (RI)
Inner
Outer
G
DAPI + Aspa RI
DAPI
Aspa RI
K8

Supplementary Fig. 3 *Aspa* transcripts are expressed during MC differentiation.

(A) Transcript schematic of mouse *Aspa* gene locus from Ensembl database. Mouse *Aspa* gene locus encodes 6 different transcripts: *Aspa*-201, 202…, 206. Rectangular boxes represent exons. Boxes with solid colors indicate protein coding region. Bold lines indicate the target sequences of the smFISH probes which recognize mRNA transcripts *Aspa*-201/203/205/206 (mRNA probe #1, red), mRNA transcripts *Aspa*-201/206 (mRNA probe #2, red), and the retained intron (RI) transcript *Aspa*-202 (cyan).

(B) Scatter plots indicating the expression levels of *Aspa* transcripts in single MCs along the pseudotime of MC differentiation.

(C) Scatter plot showing the absolute read counts aligned to the retained intron of *Aspa*-202 in single MCs along the pseudotime of MC differentiation.

(D-F) Spatial correlation of *Aspa* mRNA and *Aspa* RI smFISH signal intensity in individual MCs from Figure 2D as indicated by Pearson’s correlation and Mander's split coefficients. Boxplots show median interquartile range (IQR), and whiskers extending to 1.5 x IQR. Data were collected from 9 touch domes across 3 mice. N (Inner) = 88; N (Outer) = 92.

(G) Representative confocal optical section of smFISH displaying the extrachromosomal localization of *Aspa* RI (red) transcripts in a dissociated mouse MC expressing K8 (green). Chromosome/DNA, DAPI (blue). Arrow, MC expressing *Aspa* RI. Scale bar, 10µm.

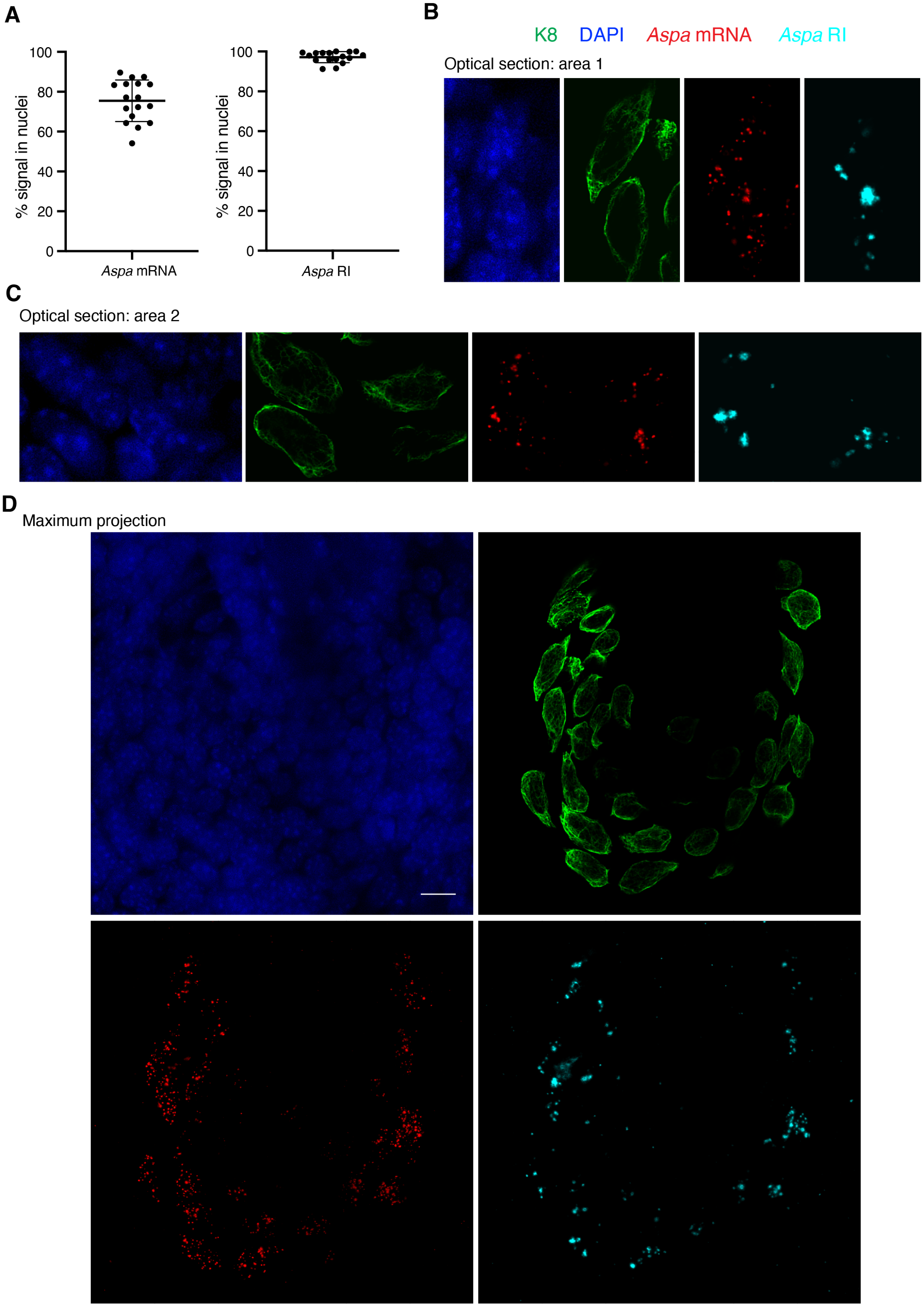
A
100
80
60
40
20
0
% signal in nuclei
Aspa mRNA
Aspa RI
B
K8
DAPI
Aspa mRNA
Aspa RI
Optical section: area 1
C
Optical section: area 2
D
Maximum projection

Supplementary Fig. 4 Retained intron transcript *Aspa*-202 is co-expressed with *Aspa* mRNA transcripts in single MCs and forms nuclear condensates.

(A) Scatter plot showing the proportion of nuclear *Aspa* mRNA and RI smFISH signal in single MCs, analyzed in Imaris from z-stacked images. Data are presented as mean ± SD (N = 17).

(B-D) Separate channel images for Figure 2D. Scale bar, 10µm.

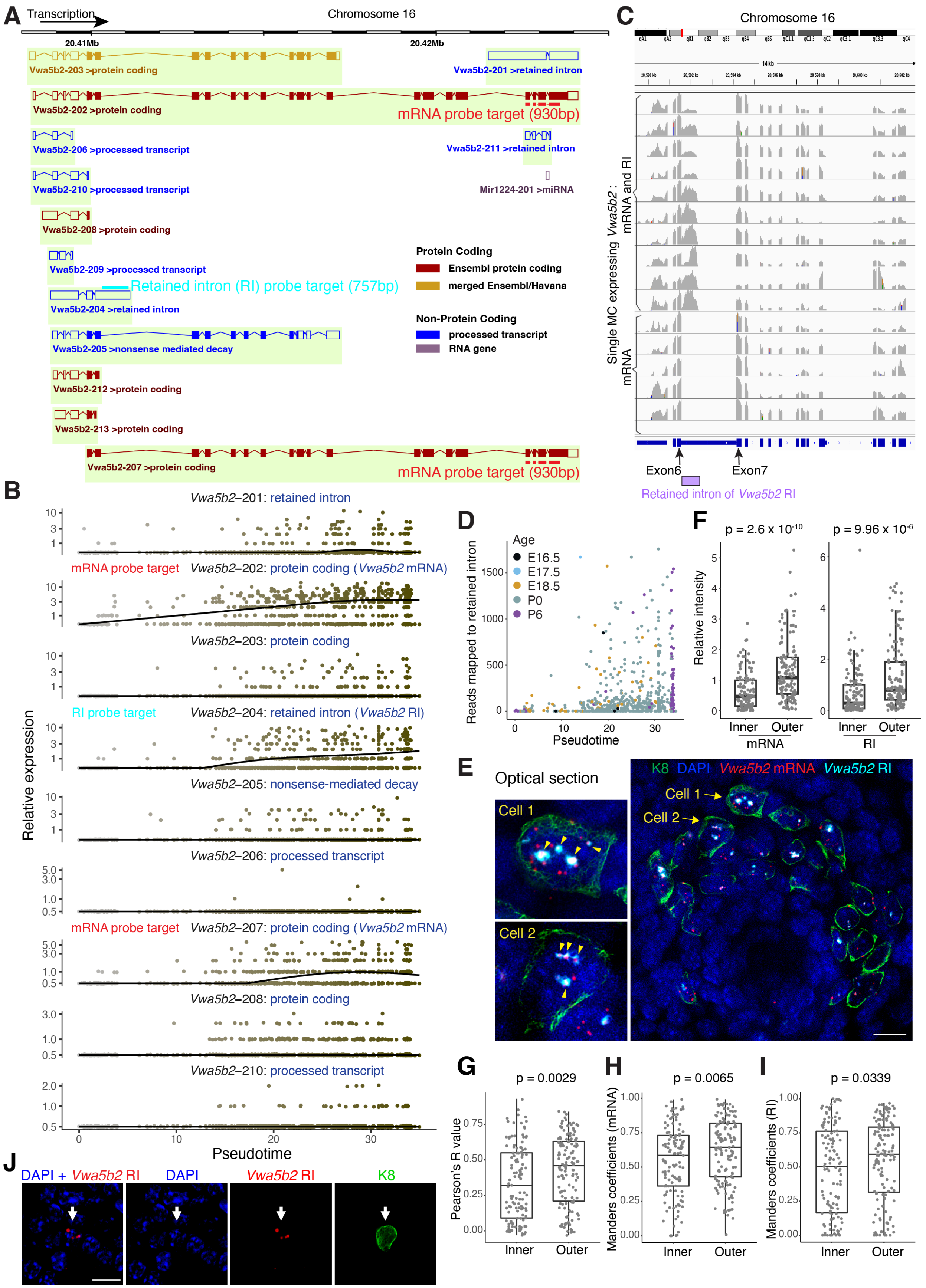
A
Transcription
Chromosome 16
20.41Mb
20.42Mb
Vwa5b2-203 >protein coding
Vwa5b2-201 >retained intron
Vwa5b2-202 >protein coding
mRNA probe target (930bp)
Vwa5b2-206 >processed transcript
Vwa5b2-211 >retained intron
Vwa5b2-210 >processed transcript
Mir1224-201 >miRNA
Vwa5b2-208 >protein coding
Vwa5b2-209 >processed transcript
Retained intron (RI) probe target (757bp)
Vwa5b2-204 >retained intron
Vwa5b2-205 >nonsense mediated decay
Vwa5b2-212 >protein coding
Vwa5b2-213 >protein coding
Vwa5b2-207 >protein coding
mRNA probe target (930bp)
Protein Coding
Ensembl protein coding
merged Ensembl/Havana
Non-Protein Coding
processed transcript
RNA gene
C
Chromosome 16
Single MC expressing Vwa5b2 : mRNA and RI
mRNA
Exon6
Exon7
Retained intron of Vwa5b2 RI
B
Relative expression
Vwa5b2–201: retained intron
mRNA probe target
Vwa5b2–202: protein coding (Vwa5b2 mRNA)
Vwa5b2–203: protein coding
RI probe target
Vwa5b2–204: retained intron (Vwa5b2 RI)
Vwa5b2–205: nonsense-mediated decay
Vwa5b2–206: processed transcript
mRNA probe target
Vwa5b2–207: protein coding (Vwa5b2 mRNA)
Vwa5b2–208: protein coding
Vwa5b2–210: processed transcript
Pseudotime
D
Age
E16.5
E17.5
E18.5
P0
P6
Reads mapped to retained intron
Pseudotime
F
p = 2.6 x 10-10
p = 9.96 x 10-6
Relative intensity
Inner
Outer
mRNA
Inner
Outer
RI
E
Optical section
Cell 1
Cell 2
K8 DAPI Vwa5b2 mRNA Vwa5b2 RI
Cell 1
Cell 2
G
p = 0.0029
Pearson's R value
Inner
Outer
H
p = 0.0065
Manders coefficients (mRNA)
Inner
Outer
I
p = 0.0339
Manders coefficients (RI)
Inner
Outer
J
DAPI + Vwa5b2 RI
DAPI
Vwa5b2 RI
K8

Supplementary Fig. 5 The *Vwa5b2*-204 retained intron (RI) transcript forms extrachromosomal nuclear condensates in differentiating MCs that co-localize with *Vwa5b2* mRNA transcripts.

(A) Transcript schematic of mouse *Vwa5b2* gene locus from Ensembl database. Mouse *Vwa5b2* gene locus encodes 13 different transcripts: *Vwa5b2*-201, 202…, 213. Rectangular boxes represent exons. Boxes with solid colors indicate protein-coding region. Bold lines indicate the target sequences of the smFISH probes which recognize mRNA transcripts *Vwa5b2*-202/207 (red) and the RI transcript *Vwa5b2*-204 (cyan).

(B) Scatter plots indicating the expression levels of *Vwa5b2* transcripts in single MCs along the pseudotime of MC differentiation.

(C) IGV-generated plot of sequencing reads aligned to *Vwa5b2* gene locus indicating RI transcripts in selected MCs.

(D) Scatter plot showing the read counts aligned to the retained intron of *Vwa5b2*-204 in single MC along the pseudotime of differentiation.

(E) Representative confocal optical section of whole mount smFISH displaying the expression of *Vwa5b2* mRNA (red) and *Vwa5b2* RI (cyan) transcripts in neonatal mouse epidermis. MCs, K8 immunostaining (green). Cell nuclei, DAPI (blue). Arrowhead indicates colocalization of *Vwa5b2* mRNA and *Vwa5b2* RI. Scale bar, 10µm.

(F) Boxplot showing the smFISH signal intensity of the target transcripts per cell in inner and outer touch dome MCs. Boxplots show median, interquartile range (IQR), and whiskers extending to 1.5 x IQR. Data were collected from 10 touch domes across 3 mice. N (Inner) = 121; N (Outer) = 137.

(G-I) Spatial correlation of *Vwa5b2* mRNA and *Vwa5b2* RI smFISH signal intensity in individual MCs as indicated by Pearson's correlation and Mander's split coefficients. Boxplots show median, interquartile range (IQR), and whiskers extending to 1.5 x IQR. Data were collected from 10 touch domes across 3 mice. N (Inner) = 121; N (Outer) = 137.

(J) Representative confocal optical section of smFISH displaying the extrachromosomal localization of *Vwa5b2* RI (red) transcripts in a dissociated mouse MC expressing K8 (green). Chromosome/DNA, DAPI (blue). Arrow, MC expressing *Vwa5b2* RI. Scale bar, 10µm.

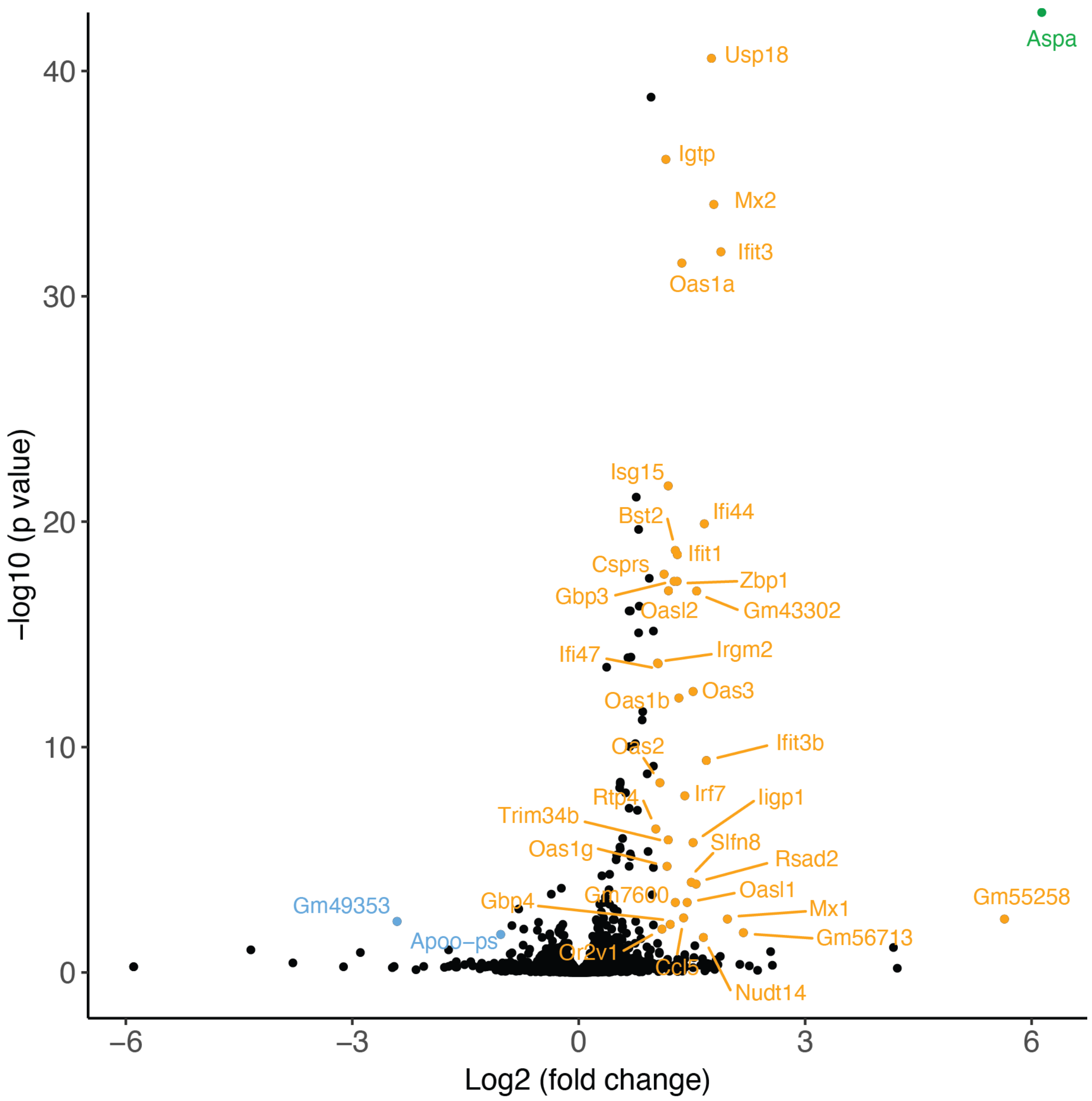

Supplementary Fig. 6 *Aspa*-202 retained intron transcript does not induce a global reduction in RNA expression. Volcano plot showing differentially expressed genes upon Aspa-202 overexpression compared to empty vector control. Upregulated genes (adjusted p-value < 0.05 and $\log_2$(fold change) >1) are shown in orange; downregulated genes (adjusted p-value < 0.05 and $\log_2$(fold change) < -1) are shown in blue. *Aspa* shown in green, appears upregulated due to read mapping from the overexpressed retained intron transcript.

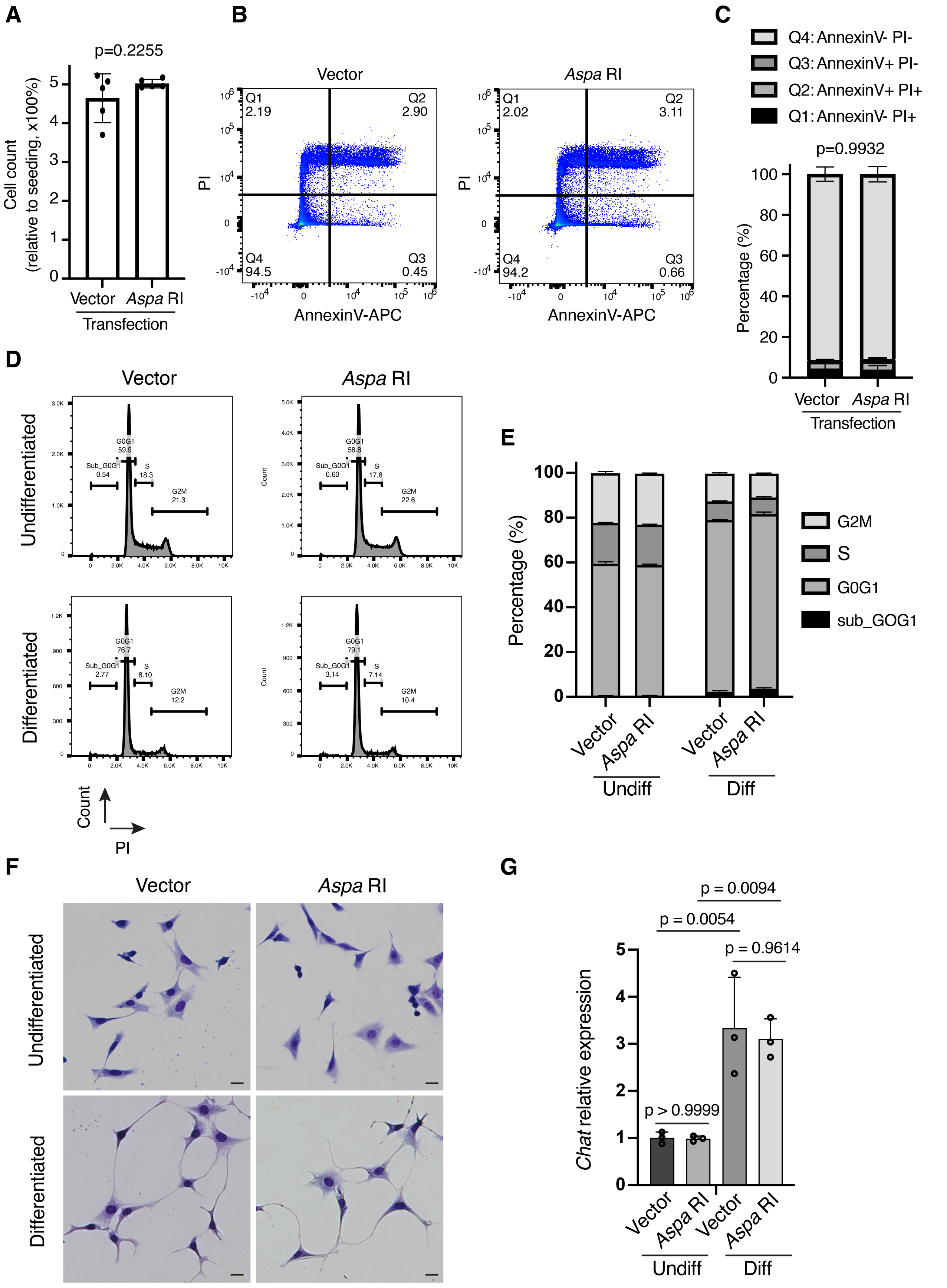

A
p=0.2255
Cell count
(relative to seeding, x100%)
Vector
Aspa RI
Transfection
B
Vector
Aspa RI
Q1
2.19
Q2
2.90
Q4
94.5
Q3
0.45
Q1
2.02
Q2
3.11
Q4
94.2
Q3
0.66
PI
AnnexinV-APC
C
Q4: AnnexinV- PI-
Q3: AnnexinV+ PI-
Q2: AnnexinV+ PI+
Q1: AnnexinV- PI+
p=0.9932
Percentage (%)
Vector
Aspa RI
Transfection
D
Vector
Aspa RI
Undifferentiated
Differentiated
G0G1
59.9
Sub_G0G1
0.54
S
18.3
G2M
21.3
G0G1
58.8
Sub_G0G1
0.60
S
17.8
G2M
22.6
G0G1
76.7
Sub_G0G1
2.77
S
8.10
G2M
12.2
G0G1
79.1
Sub_G0G1
3.14
S
7.14
G2M
10.4
Count
PI
E
Percentage (%)
G2M
S
G0G1
sub_GOG1
Vector
Aspa RI
Vector
Aspa RI
Undiff
Diff
F
Vector
Aspa RI
Undifferentiated
Differentiated
G
p = 0.0094
p = 0.0054
p = 0.9614
p > 0.9999
Chat relative expression
Vector
Aspa RI
Vector
Aspa RI
Undiff
Diff

Supplementary Fig.7 *Aspa*-202 retained intron transcript does not impact cell proliferation, apoptosis, cell cycle or neuronal differentiation of HT-22 cells.

(A) Bar chart showing quantification of cell count 48 hours post-transfection, showing no difference in proliferation after overexpression of *Aspa* RI. Data are presented as mean ± SD (N = 5).

(B) Representative flow cytometry dot plot showing cell apoptosis (Q2 + Q3) and necrosis (Q1), assessed by PI and Annexin V staining.

(C) Bar chart showing the quantification of apoptotic (Q2 + Q3) and necrotic (Q1) cells based on data from B). Data are presented as mean ± SD (N = 4).

(D) Representative flow cytometry histogram depicting cell cycle distribution in both undifferentiated and differentiated HT-22 cells, assessed by PI staining.

(E) Bar chart showing the quantitative analysis of cell cycle phase distribution from (D). Undiff, undifferentiated; Diff, differentiated. Data are presented as mean ± SD (N = 3).

(F) Representative Giemsa-stained images displaying the effect of *Aspa* RI expression on the cell morphology of both undifferentiated and differentiated HT-22 cells. Scale bar, 20 μm.

(G) Bar chart showing relative expression of *Chat* mRNA normalized to *Rpl13a* after *Aspa* RI overexpression in both undifferentiated (Undiff) and differentiated (Diff) HT-22 cells. Data are presented as mean ± SD (N = 3).

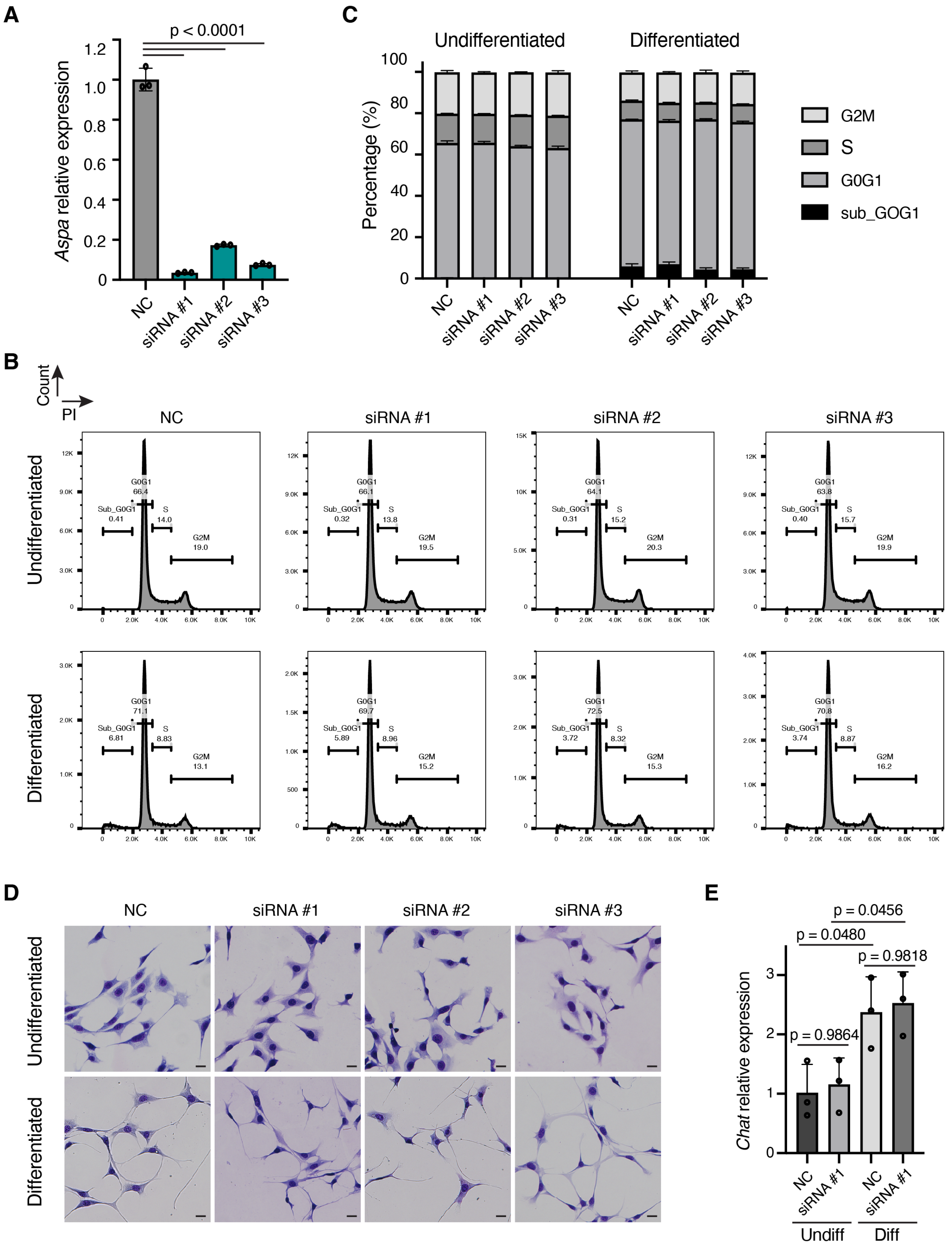

A
p < 0.0001
Aspa relative expression
1.2
1.0
0.8
0.6
0.4
0.2
0
NC
siRNA #1
siRNA #2
siRNA #3
C
Undifferentiated
Differentiated
Percentage (%)
100
80
60
40
20
0
G2M
S
G0G1
sub_GOG1
B
Count
PI
NC
siRNA #1
siRNA #2
siRNA #3
Undifferentiated
Differentiated
Sub_G0G1 0.41
G0G1 66.4
S 14.0
G2M 19.0
Sub_G0G1 0.32
G0G1 66.1
S 13.8
G2M 19.5
Sub_G0G1 0.31
G0G1 64.1
S 15.2
G2M 20.3
Sub_G0G1 0.40
G0G1 63.8
S 15.7
G2M 19.9
Sub_G0G1 6.81
G0G1 71.1
S 8.83
G2M 13.1
Sub_G0G1 5.89
G0G1 69.7
S 8.96
G2M 15.2
Sub_G0G1 3.72
G0G1 72.5
S 8.32
G2M 15.3
Sub_G0G1 3.74
G0G1 70.8
S 8.87
G2M 16.2
D
NC
siRNA #1
siRNA #2
siRNA #3
Undifferentiated
Differentiated
E
p = 0.0456
p = 0.0480
p = 0.9818
p = 0.9864
Chat relative expression
3
2
1
0
NC
siRNA #1
NC
siRNA #1
Undiff
Diff

Supplementary Fig. 8 *Aspa* knockdown does not impact HT-22 cell cycle or neuronal differentiation.

(A) Bar chart showing relative expression of *Aspa* mRNA normalized to *Rpl13a* in HT-22 cells transfected *Aspa* or negative control siRNAs. Data are presented as mean ± SD (N = 3), p values by ANOVA. NC, negative control non-targeting siRNA.

(B) Representative flow cytometry histogram depicting cell cycle distribution, assessed by PI staining.

(C) Bar chart showing the quantitative analysis of cell cycle phase distribution from (B). Data are presented as mean ± SD (N = 3).

(D) Representative Giemsa-stained images displaying the cell morphology of differentiated HT-22 cells compared to undifferentiated control. Scale bar, 20 μm.

(E) Bar chart showing relative expression of *Chat* mRNA normalized to *Rpl13a* in HT-22 cells subjected to different treatments. Undiff, undifferentiated; Diff, differentiated. Data are presented as mean ± SD (N = 3).

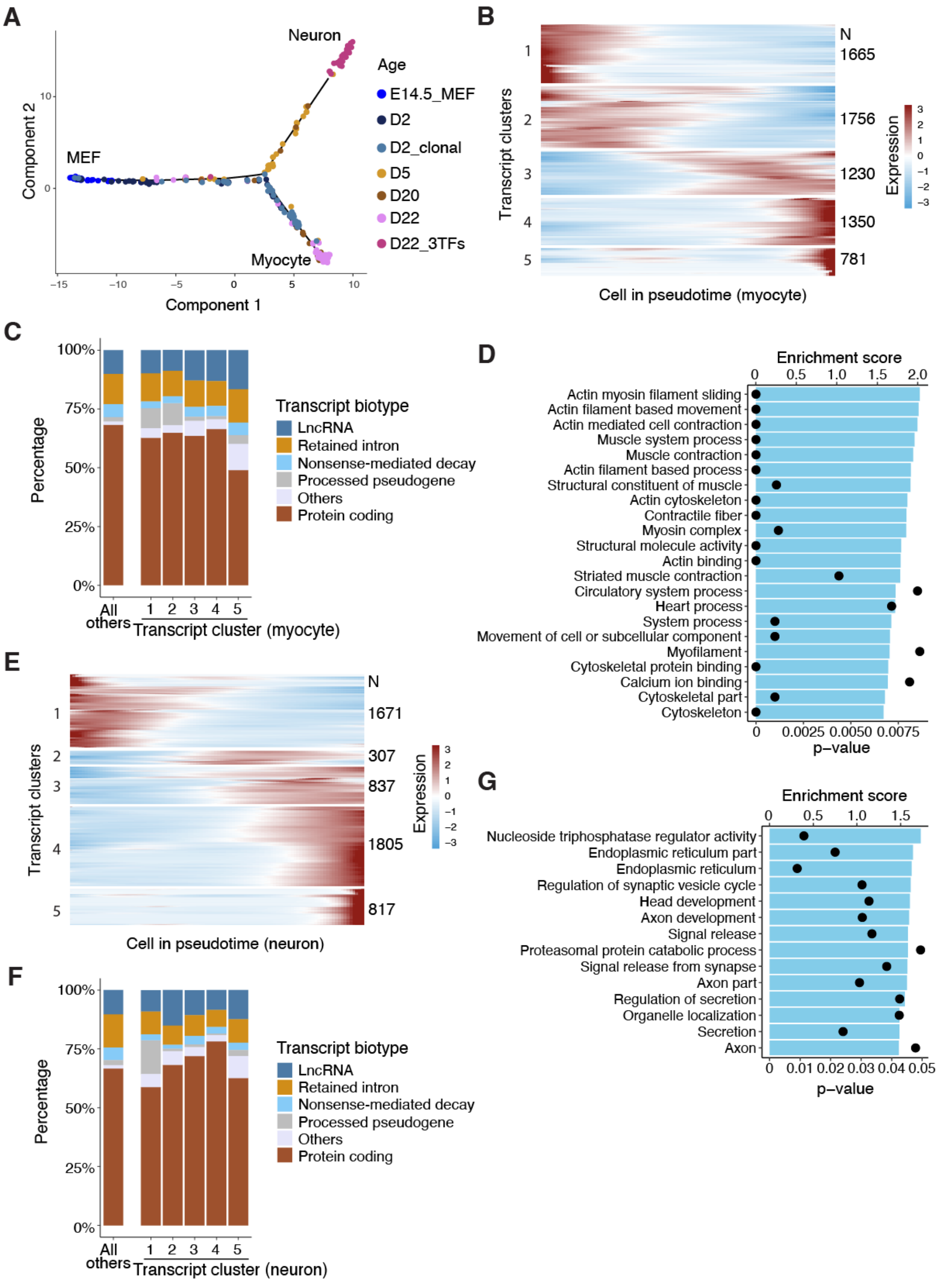
A
Neuron
MEF
Myocyte
Component 2
Component 1
Age
E14.5_MEF
D2
D2_clonal
D5
D20
D22
D22_3TFs
B
N
1665
1756
1230
1350
781
Transcript clusters
Expression
Cell in pseudotime (myocyte)
C
Percentage
All others
Transcript cluster (myocyte)
Transcript biotype
LncRNA
Retained intron
Nonsense-mediated decay
Processed pseudogene
Others
Protein coding
D
Enrichment score
Actin myosin filament sliding
Actin filament based movement
Actin mediated cell contraction
Muscle system process
Muscle contraction
Actin filament based process
Structural constituent of muscle
Actin cytoskeleton
Contractile fiber
Myosin complex
Structural molecule activity
Actin binding
Striated muscle contraction
Circulatory system process
Heart process
System process
Movement of cell or subcellular component
Myofilament
Cytoskeletal protein binding
Calcium ion binding
Cytoskeletal part
Cytoskeleton
p−value
E
N
1671
307
837
1805
817
Transcript clusters
Expression
Cell in pseudotime (neuron)
F
Percentage
All others
Transcript cluster (neuron)
Transcript biotype
LncRNA
Retained intron
Nonsense-mediated decay
Processed pseudogene
Others
Protein coding
G
Enrichment score
Nucleoside triphosphatase regulator activity
Endoplasmic reticulum part
Endoplasmic reticulum
Regulation of synaptic vesicle cycle
Head development
Axon development
Signal release
Proteasomal protein catabolic process
Signal release from synapse
Axon part
Regulation of secretion
Organelle localization
Secretion
Axon
p−value

Supplementary Fig. 9 Emergence of non-coding transcript biotypes is observed in differentiating myocytes and neurons derived in vitro from mouse embryonic fibroblasts (MEFs).

(A) Monocle-generated trajectory plot representing single cell pseudotime ordering of MEFs differentiating to myocytes and neurons. E, embryonic day; D, in vitro protocol day; D2_clonal, clonally selected source MEFs; D22_3TFs, cells transduced with three transcription factors. See methods for details.

(B and E) Scaled and centered expression heatmaps of transcripts with dynamic expression over the differentiation pseudotime from MEFs to myocytes (B) and neurons (E). Transcripts are ordered by row and cells by column. Transcripts are clustered by their expression patterns over pseudotime. Numbers of transcripts per cluster are shown.

(C and F) Bar charts showing the relative proportion of transcript biotypes in each of the pseudotime-dependent transcript clusters and in all cells. All others: 13004 all other detected transcripts with TPM>10 in more than 21 MEFs/myocytes (C) and 16308 all other detected transcripts with TPM>10 in more than 14 MEFs/neurons (F). Chi-squared test: $p < 2.2 \times 10^{-16}$ for protein coding vs. non-coding transcript numbers across all clusters, including “All others”.

(D and G) Top gene ontology terms associated with genes encoding non-coding transcript biotypes in transcript cluster 4 and 5 (highest expression in late/differentiated cells) during differentiation from MEFs to myocytes (D) and neurons (G). Bar magnitude, enrichment score; dot, p-value.

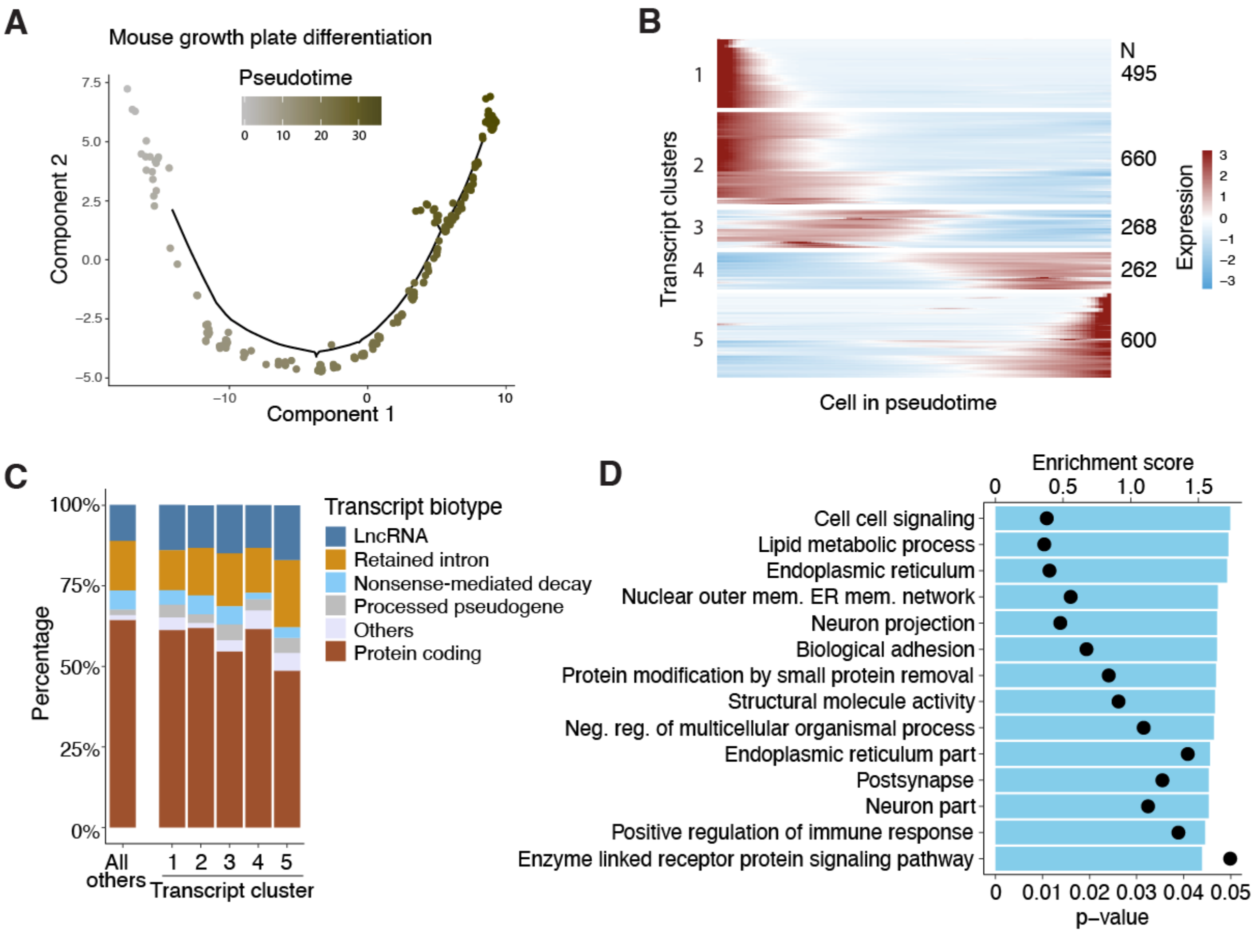

Supplementary Fig. 10 Emergence of non-coding transcript biotypes is observed in differentiating mouse growth plate.

(A) Monocle-generated trajectory plot representing differentiation pseudotime ordering of single mouse growth plate cells.

(B) Scaled and centered expression heatmap of transcripts with dynamic expression over the differentiation pseudotime of mouse growth plate. Transcripts are ordered by row and cells by column. Transcripts are clustered by their expression patterns over pseudotime. Numbers of transcripts per cluster are shown.

(C) Bar chart showing the relative proportion of transcript biotypes in each of the pseudotime-dependent transcript clusters and in all cells. All others: 21183 detected transcripts with TPM>10 in more than 7 cells. mem., membrane. Chi-squared test: $p = 1.699 \times 10^{-14}$ for protein coding vs. non-coding transcript numbers across all clusters, including “All others”.

(D) Top gene ontology terms associated with genes encoding non-coding transcript biotypes in transcript cluster 4 and 5 (highest expression in late/differentiated cells) during mouse growth plate differentiation.

Bar magnitude, enrichment score; dot, p-value. Abbreviations: mem., membrane; Neg. reg., negative regulation.

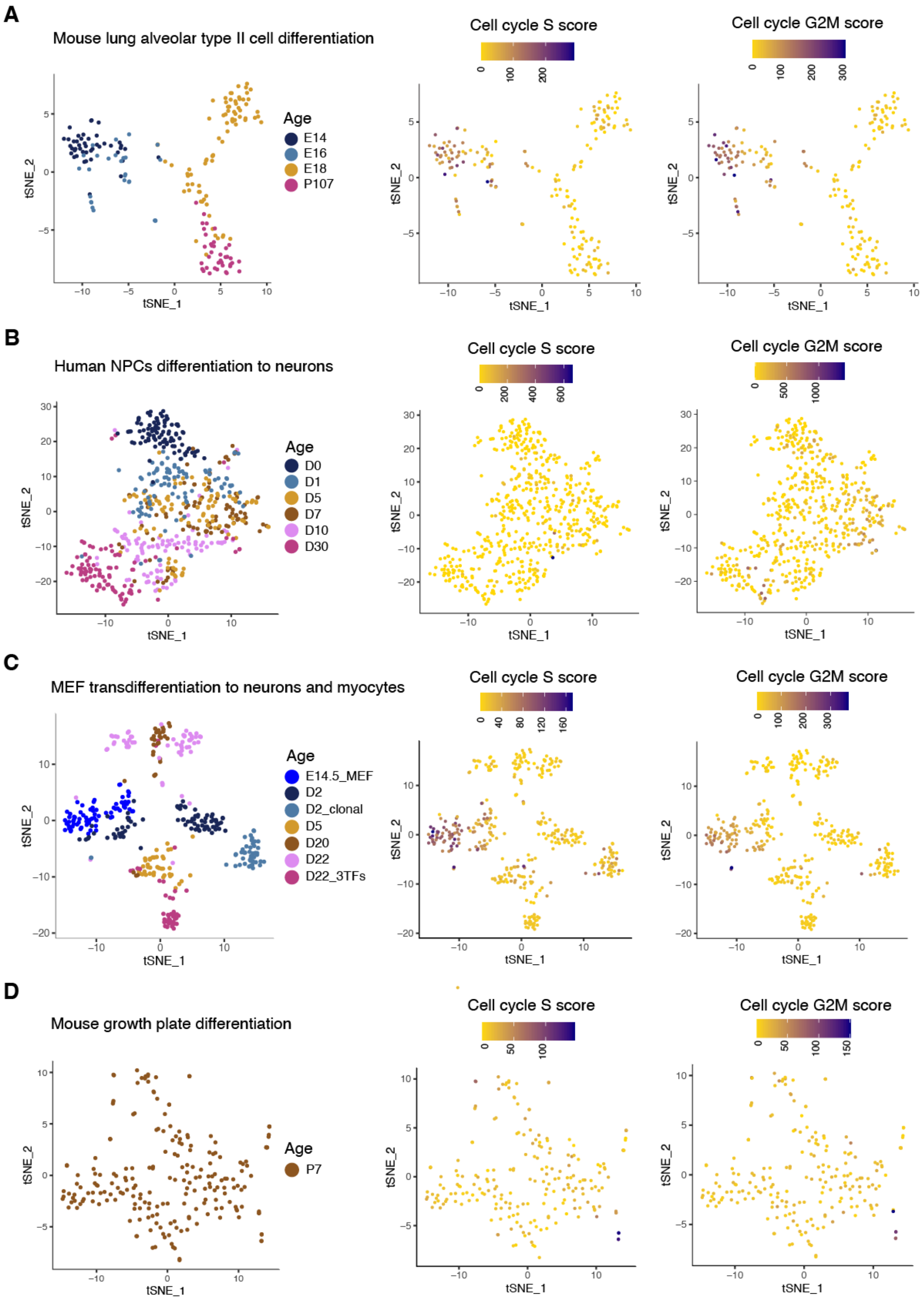
A
Mouse lung alveolar type II cell differentiation
Age
E14
E16
E18
P107
tSNE_1
tSNE_2
Cell cycle S score
Cell cycle G2M score
B
Human NPCs differentiation to neurons
Age
D0
D1
D5
D7
D10
D30
Cell cycle S score
Cell cycle G2M score
C
MEF transdifferentiation to neurons and myocytes
Age
E14.5_MEF
D2
D2_clonal
D5
D20
D22
D22_3TFs
Cell cycle S score
Cell cycle G2M score
D
Mouse growth plate differentiation
Age
P7
Cell cycle S score
Cell cycle G2M score

Supplementary Fig. 11 Cell cycle genes are restricted to early differentiation stages in multiple cell types.

tSNE plots showing sampling time points (left) and feature plots showing cell cycle score (middle, S phase; right, G2M phase) in single cell differentiation of mouse ATII cells (A), human neurons from NPCs (B), myocytes and neurons from MEFs (C), and mouse growth plate cells (D).

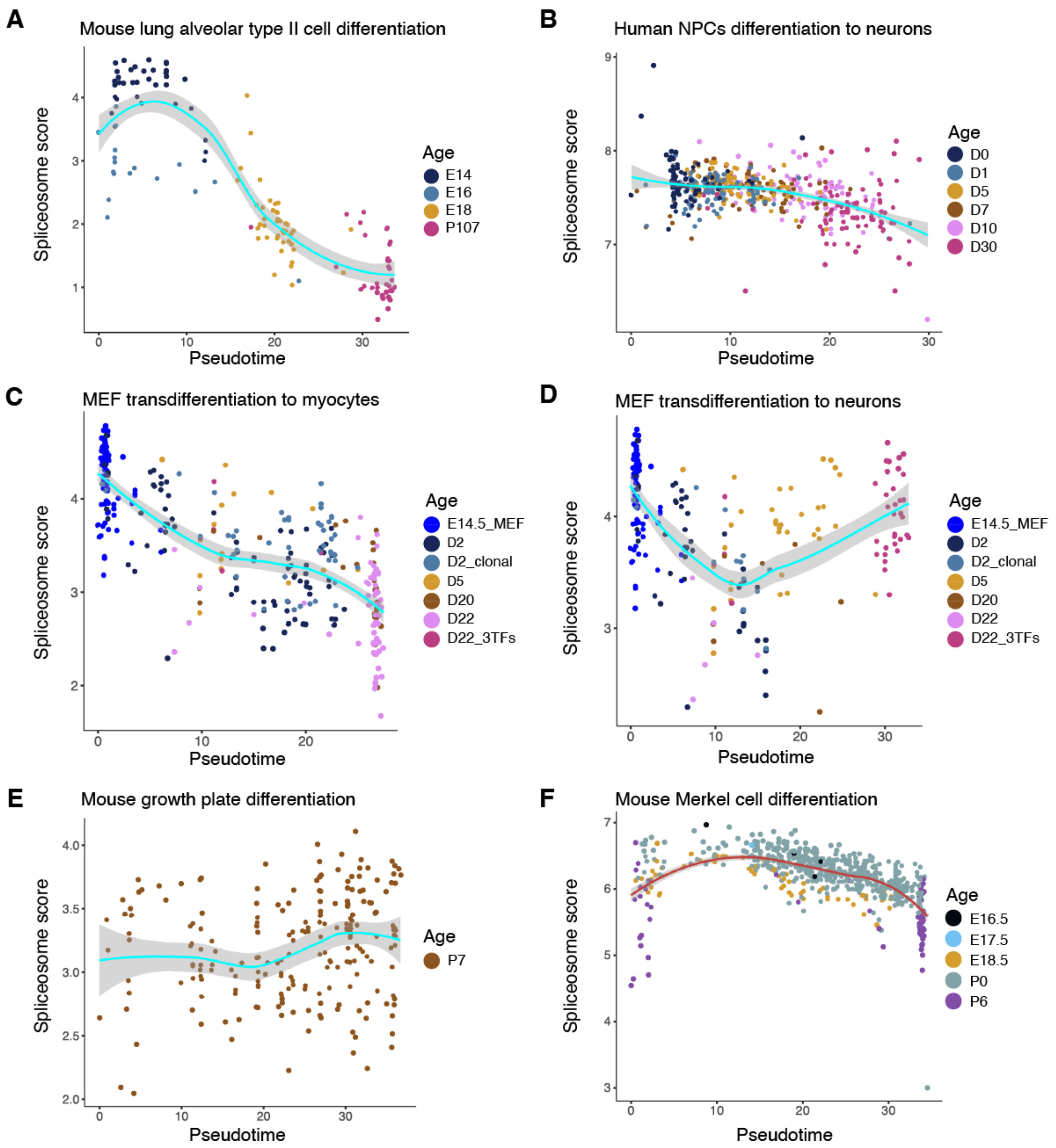

Supplementary Fig. 12 Splicing factor expression varies inconsistently during differentiation in different cell types.

Scatter plots indicating the expression levels of spliceosome genes (represented by spliceosome score: total TPM of spliceosome genes, log2 transformed) in single cells along the pseudotime of mouse ATII cell differentiation (A), human neurons differentiation from NPCs (B), differentiation of MEFs to myocytes (C) and neurons (D), differentiation of mouse growth plate cells (E), and differentiation of mouse MCs (F). The line and shading show LOESS regression and 95% confidence interval of spliceosome scores.

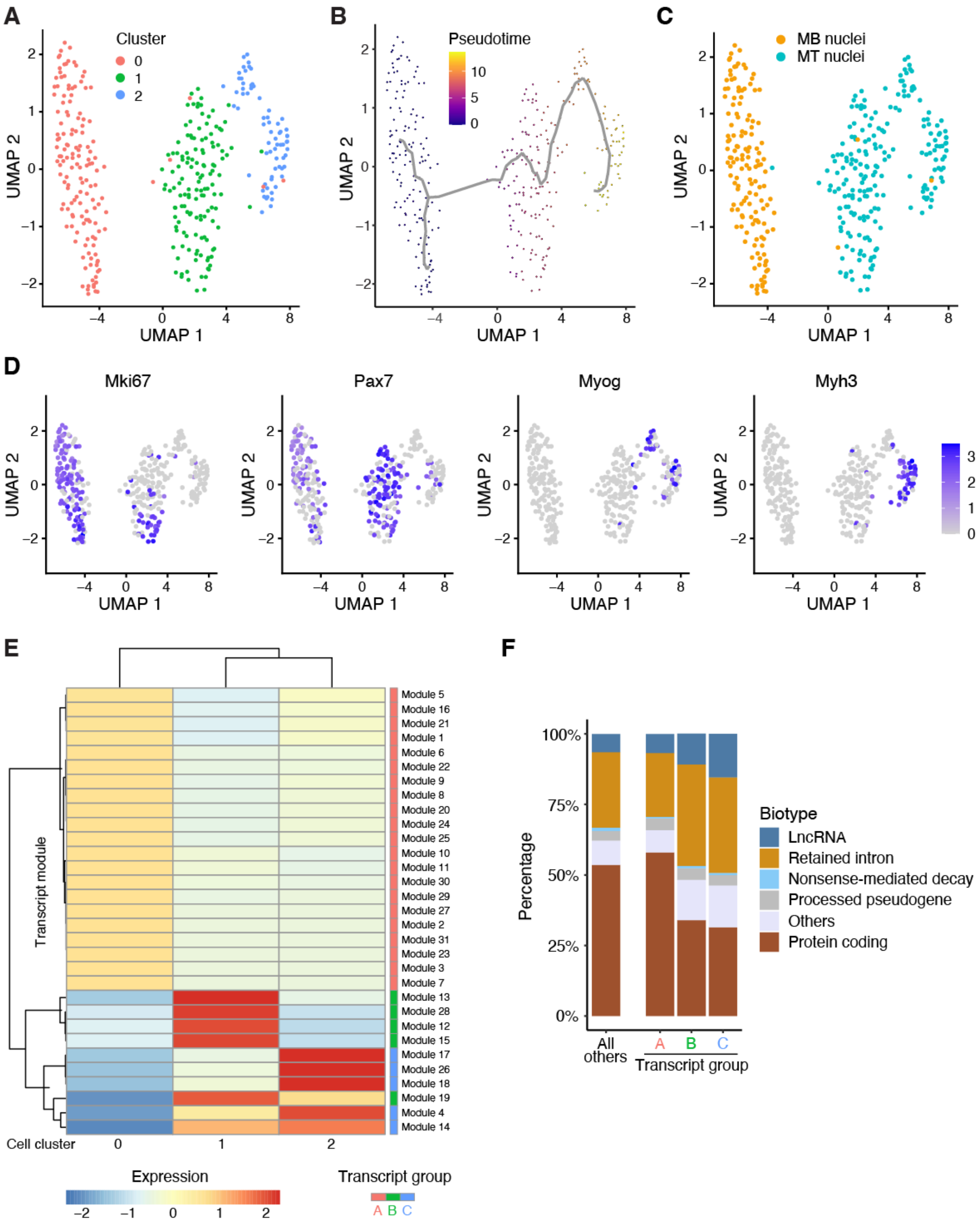

Supplementary Fig. 13 Emergence of non-coding transcript biotypes is observed in myotubes differentiated from C2C12 myoblasts as detected by long-read single-nuclei RNA sequencing.

(A) UMAP plot displaying three cell clusters identified by differential gene expression analysis.

(B) Same UMAP plot as A showing the differentiation pseudotime identified by trajectory analysis.

(C) Same UMAP plot as A labeled with source cell type, C2C12 myoblasts (MB) or differentiating myotubes (MT).

(D) Feature plots indicating the expression levels of *Mki67*, *Pax7*, *Myog*, *Myh3* genes in single cells.

(E) Scaled and centered expression heatmap of transcript modules with differential expression over the differentiation pseudotime of C2C12 cells. Transcript modules are ordered by row and cell clusters by column. Modules were assigned to transcript groups A, B or C based on if their highest transcript expression was observed in cell cluster 0, 1, or 2.

(F) Bar chart showing the relative proportion of transcript biotypes in each of the transcript groups. All others: 20562 all other detected transcripts with TPM>10 in more than 1 cells. Chi-squared test: $p = 5.278 \times 10^{-13}$ for protein coding vs. non-coding transcript numbers across all groups, including "All others".

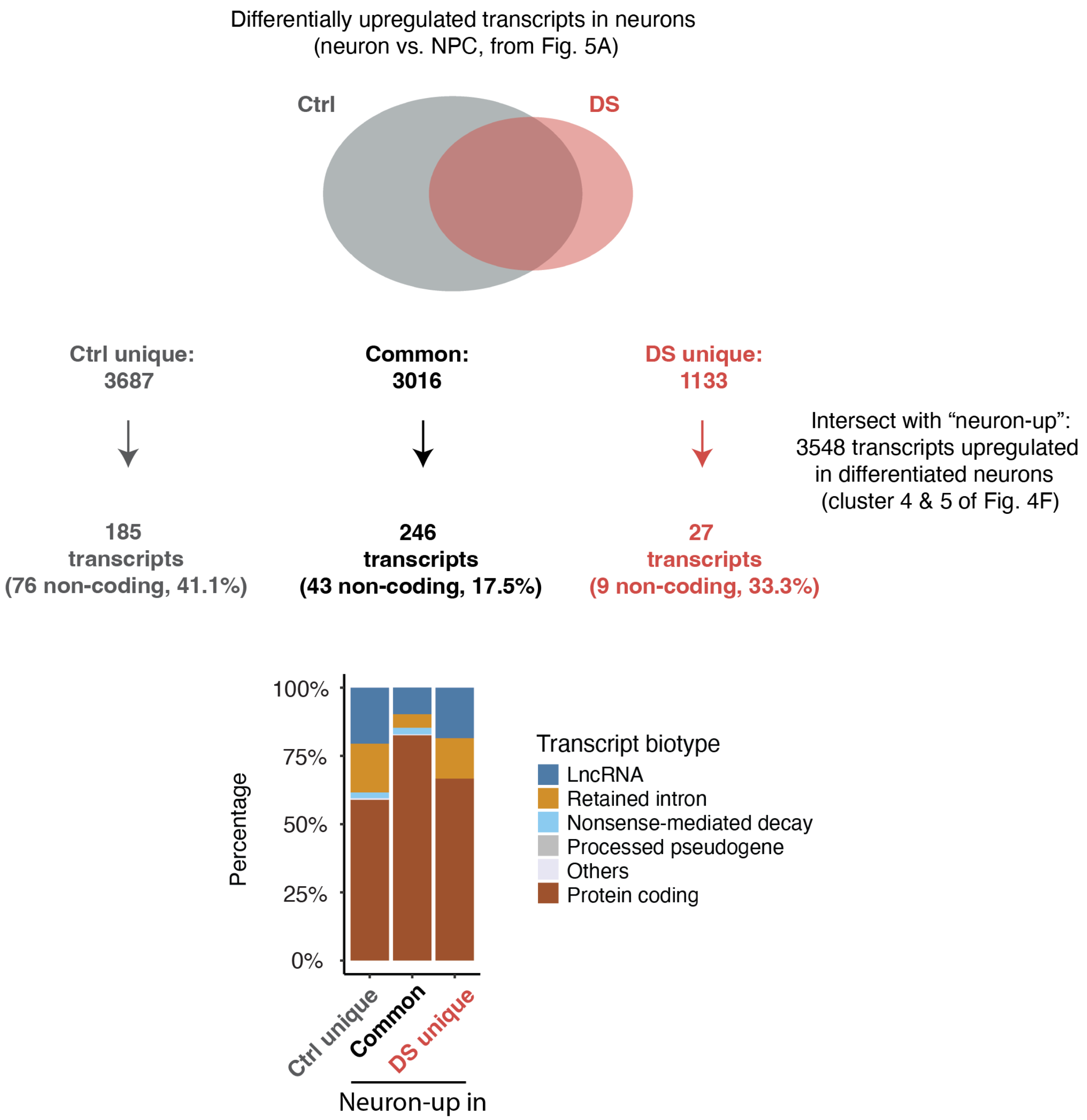

Supplementary Fig. 14 Emergence of neuronal differentiation associated non-coding transcript biotypes is diminished in differentiating Down syndrome (DS) neurons. Schematic illustration showing the identification of transcripts that are both upregulated during terminal differentiation of human *in vitro*-cultured neurons (from Fig. 4F), and are differentially upregulated in neurons relative to NPCs derived from control (ctrl) and DS iPSCs (control unique, DS unique and common to both control and DS; from Fig. 5A). **Bottom:** Bar chart showing the relative proportion of transcript biotypes for these transcripts, categorized as control unique, DS unique, or commonly upregulated in both control and DS.

Legends for Supplementary Videos 1 and 2

Supplementary Video 1. 3D visualization of whole mount smFISH showing the expression of *Aspa* mRNA (red) and *Aspa* RI (cyan) transcripts in neonatal mouse epidermis, corresponding to area 1, Figure 2D. MCs, K8 immunostaining (green). Cell nuclei, DAPI (blue).

Supplementary Video 2. 3D visualization of whole mount smFISH showing the expression of *Vwa5b2* mRNA (red) and *Vwa5b2* RI (cyan) transcripts in neonatal mouse epidermis, corresponding to Supplementary Figure 5E. MCs, K8 immunostaining (green). Cell nuclei, DAPI (blue).
